# A Transcriptomic Approach to Understanding the Combined Impacts of Supra-Optimal Temperatures and CO_2_ Revealed Different Responses in the Polyploid *Coffea arabica* and Its Diploid Progenitor *C. canephora*

**DOI:** 10.3390/ijms22063125

**Published:** 2021-03-18

**Authors:** Isabel Marques, Isabel Fernandes, Octávio S. Paulo, Fernando C. Lidon, Fábio M. DaMatta, José C. Ramalho, Ana I. Ribeiro-Barros

**Affiliations:** 1 Plant-Environment Interactions and Biodiversity Lab (PlantStress&Biodiversity), Forest Research Centre (CEF), Instituto Superior de Agronomia (ISA), Universidade de Lisboa, 2784-505 Oeiras and Tapada da Ajuda, 1349-017 Lisboa, Portugal; 2 Computational Biology and Population Genomics Group, Centre for Ecology, Evolution and Environmental Changes (cE3c), Faculdade de Ciências, Universidade de Lisboa, 1749-016 Lisboa, Portugal; isabelcmoniz@gmail.com (I.F.); ofpaulo@fc.ul.pt (O.S.P.); 3 GeoBioSciences, GeoTechnologies and GeoEngineering (GeoBioTec), Faculdade de Ciências e Tecnologia (FCT), Universidade NOVA de Lisboa (UNL), 2829-516 Monte de Caparica, Portugal; fjl@fct.unl.pt; 4 Departamento de Biologia Vegetal, Universidade Federal Viçosa (UFV), Viçosa 36570-090, MG, Brazil; fdamatta@ufv.br

**Keywords:** climate changes, coffee, elevated air (CO_2_), functional analysis, high temperatures, leaf RNAseq, polyploidy, warming

## Abstract

Understanding the effect of extreme temperatures and elevated air (CO_2_) is crucial for mitigating the impacts of the coffee industry. In this work, leaf transcriptomic changes were evaluated in the diploid *C. canephora* and its polyploid *C. arabica*, grown at 25 °C and at two supra-optimal temperatures (37 °C, 42 °C), under ambient (aCO_2_) or elevated air CO_2_ (eCO_2_). Both species expressed fewer genes as temperature rose, although a high number of differentially expressed genes (DEGs) were observed, especially at 42 °C. An enrichment analysis revealed that the two species reacted differently to the high temperatures but with an overall up-regulation of the photosynthetic machinery until 37 °C. Although eCO_2_ helped to release stress, 42 °C had a severe impact on both species. A total of 667 photosynthetic and biochemical related-DEGs were altered with high temperatures and eCO_2_, which may be used as key probe genes in future studies. This was mostly felt in *C. arabica*, where genes related to ribulose-bisphosphate carboxylase (RuBisCO) activity, chlorophyll a-b binding, and the reaction centres of photosystems I and II were down-regulated, especially under 42°C, regardless of CO_2_. Transcriptomic changes showed that both species were strongly affected by the highest temperature, although they can endure higher temperatures (37 °C) than previously assumed.

## 1. Introduction

Temperature and carbon dioxide (CO_2_) are major drivers of climate change affecting global crop production [1,2,3]. It is therefore not surprising that many crop improvement programs focus on the development of climate-smart crops resilient to climate change [4]. Yet, breeding for tolerance to a single stress may be risky because in nature plants respond to simultaneous stresses, and increasing tolerance to one stress may be at the expense of resilience to another [5,6]. For instance, high temperatures cause physiological, biochemical, and molecular changes affecting key biological processes, such as photosynthesis, by reducing electron transport, NADPH and ATP synthesis, and ribulose-bisphosphate carboxylase (RuBisCO) activity while increasing the production of H_2_O_2_ [7,8,9]. At the other extreme, elevated CO_2_ (eCO_2_) was found to improve the physiological status and to mitigate the adverse effects of high temperatures in different species, increasing photosynthesis in most C3 plants [10,11,12,13,14]. Therefore, it is of utmost importance to identify the response pathways linked to several environmental changes, to best optimize crop improvement under the future estimated climate conditions.

Coffee is among the most important agricultural products [15]. It is produced in about 80 tropical countries, with an annual production of around nine million tons of green beans and is estimated to involve 125 million people in its entire chain of value, with many smallholder farmers whose livelihoods are supported by this crop [16,17,18]. The *Coffea* genus comprises at least 125 species, although only two dominate the coffee trade: the allotetraploid *C. arabica* L. (Arabica coffee; 2n = 4x = 44) and one of its diploid ancestors, *C. canephora* Pierre ex A. Froehner (Robusta coffee; 2n = 2x = 22), with the former contributing around 60–65% of world production [19]. *Coffea arabica* is thought to be originated from a single natural polyploidization event between *C. canephora* and *C. eugenioides* that occurred in very recent evolutionary times (<50,000 years ago [20]). The quite low levels of genetic variation found in *C. arabica* when compared to its diploid progenitors are a concern in the context of environmental changes regarding the sustainability of this crop [20]. The predicted increase in global temperature could be catastrophic to *C. arabica* yields and quality, since some works consider this species more sensitive to elevated temperatures than *C. canephora* [15], with impacts being already felt on field plantations [21,22]. In fact, classical studies suggested that coffee photosynthesis would be particularly sensitive to temperatures above 20–25 °C (e.g., [23,24,25]). It was accepted that temperature averages above 23 °C accelerated the development and ripening of *C. arabica* fruits, often leading to the loss of coffee quality [26]. Continuous exposure to temperatures as high as 30 °C may restrain growth and can induce abnormalities such as the yellowing of leaves and growth of tumors at the base of the stem [27]. A relatively high temperature during blossoming, especially if associated with a prolonged dry season, may cause the abortion of flowers [26]. Nevertheless, selected cultivars under intensive management conditions have allowed *C. arabica* plantations to be spread to marginal regions with average temperatures as high as 24–25 °C, with satisfactory yields, such as in the northeast of Brazil [28,29]. Furthermore, some experiments reported that if temperature rises gradually (from 24 °C up to 33–35 °C) and considering a sufficiently long acclimation time, *C. arabica* plants can increase their photosynthesis up to temperatures around 30 °C, displaying the same efficiency as observed at 24 °C [28]. Also, some genotypes can maintain full photosynthetic functioning at temperatures as high as 35 °C [15] or even up to 39 °C for a short number of days [14,30]. The maintenance of high photosynthetic efficiency suggests the absence of negative effects on the photosynthetic structures, which agrees to some extent with the absence of membrane permeability changes after a short-time exposure up to 50 °C, as found in *C. arabica* [31]. For *C. canephora*, the optimum annual mean temperature ranges from 22 to 26 °C [32], or from 24 to 30 °C [33], but high temperatures can still be harmful, especially if the air is dry [34]. In this context, several breeding initiatives are being developed to adapt coffee crops to these environmental changes, looking back to genotypes that can be used to develop new resistant varieties while maintaining the highest cup quality (https://worldcoffeeresearch.org/, accessed on 12 January 2021).

In the last 20 years, a considerable amount of research has been devoted to environmental coffee physiology focusing on water relations and drought tolerance mechanisms, but little is still known on the mechanisms of tolerance to unfavorable temperatures, namely transcriptional and metabolic differences in response to warmer temperatures (see [35]). For instance, it has been observed that in *Coffea*, eCO_2_ promotes a high plant vigor [13] and even crop yield [36], while improving tolerance to drought [37,38,39] and supra-optimal temperatures [14,37,40,41], with positive impacts on the physical and chemical traits of the coffee beans under supra-optimal temperatures, contributing to preserve its quality [42]. We previously showed that eCO_2_ causes significant changes in the transcriptomic responses of *C. arabica* and *C. canephora*, with differentially expressed genes (DEGs) being much more abundant in the diploid than in *C. arabica* [43]. Functional analysis also revealed that under eCO_2_, *C. canephora* expressed DEGs associated to general biological processes and to a lower extent to abiotic stress responses while *C. arabica* showed an upregulation of genes linked to plant tolerance, oxidative stress control, aquaporins, reorganization of the lipid matrix membrane, chloroplast/thylakoid organization, and PS II repair. This suggested a protective role of eCO_2_ under stressful conditions.

In the present study we explored the transcriptome of *C. arabica* (cv. Icatu) and *C. canephora* (cv. Conilon Clone 153) leaves for a thorough understanding of how coffee plants regulate transcriptomic responses to elevated temperatures, and how eCO_2_ can mitigate some deleterious effects at the physiological and metabolic level. For this, we have used a comparative approach using two coffee genotypes grown under a control temperature of 25/20 °C (day/night) and then gradually exposed to increased temperatures up to 42/30 °C in combination with ambient CO_2_ (aCO_2_: 380 μL L^−1^) or elevated CO_2_ (eCO_2_: 700 μL L^−1^) air. This analysis is complementary to (and advances) previous studies, in which the plants from these two genotypes showed a temperature tolerance up to 37/28 °C [30], together with an increased photosynthetic potential under eCO_2_ [43], which might contribute to the relief of some negative effects imposed by harsh elevated temperatures.

## 2. Results

### 2.1. Overall Transcriptome Profiling and Mapping Statistics

Stringent quality assessment and data filtering generated an average of 26.3 million clean reads, from an average 28.3 million raw reads. Overall, a high proportion of reads were aligned to the reference genome since only an average of 3.17% of reads were not mapped (Table S1). Statistical details for each replicate are depicted in Table S1.

### 2.2. General Patterns of Gene Expression

**Figure 1:**
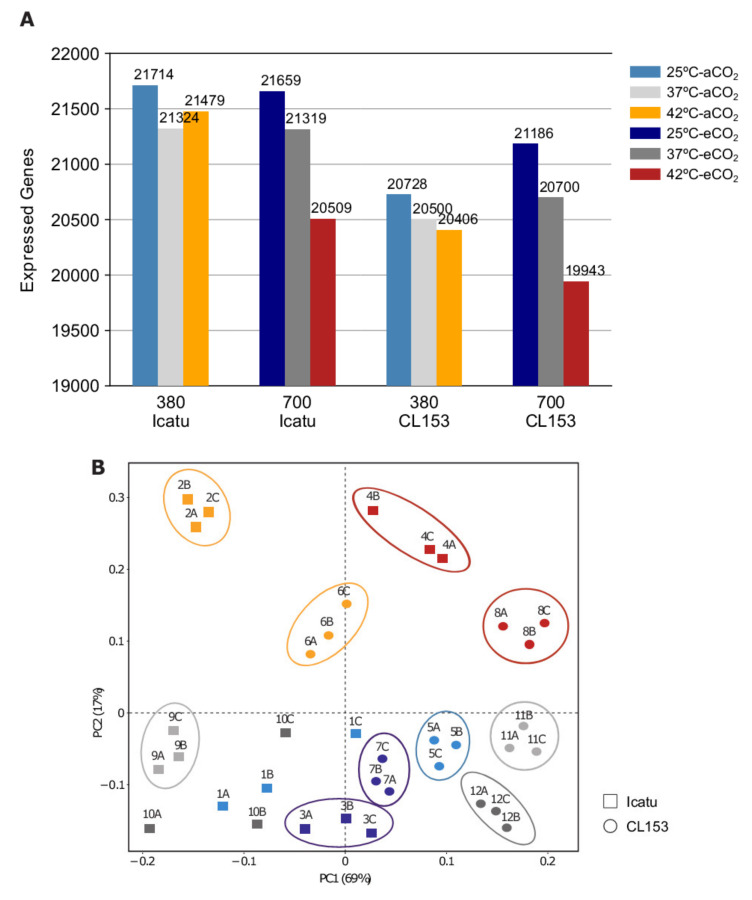
Differences in the patterns of gene expression. (**A**) Number of expressed genes in plants of *C. arabica* cv. Icatu and *C. canephora* cv. Conilon Clone 153 (CL153), grown either in ambient CO_2_ (aCO_2_, 380 μL L^−1^) or elevated CO_2_ (eCO_2_, 700 μL L^−1^) air at control temperature conditions (25/20 °C, day/night; 25 °C), a moderate supra-optimal temperature (37/28 °C; 37 °C), and an extreme supra-optimal temperature (42/30 °C; 42 °C). (**B**) Principal coordinate analysis (PCoA) of rlog transformed gene expression data generated by RNA-sequencing. Each treatment contains three biological replicates and is indicated by the colors depicted in A. The percentage of variance is indicated in each axis. Square symbols indicate Icatu, while circles indicate CL153. Detailed information is additionally given in Table S1.

The number of expressed genes varied widely between the control temperature at 25/20 °C (25 °C) and the two different supra-optimal temperatures of 37/28 °C (37 °C) and 42/30 °C (42 °C), either under 380 μL L^−1^ aCO_2_ or 700 μL L^−1^ eCO_2_. Overall, fewer genes were expressed as temperatures increased, especially in combination with eCO_2_ where the lowest number of expressed genes was observed (Figure 1A; Table S2).

The principal coordinate analysis (PCoA) revealed a stronger transcriptomic response in the two species as a result of the highest temperature (Figure 1B). In fact, PC2 separated all plants under 42 °C in the upper half of the axis from plants under 25 °C and 37 °C that were grouped below this axis (Figure 1B), irrespective of the CO_2_ condition and the species involved. PC1 was able to separate and group all Conilon Clone 153 (CL153) plants under 25 °C and 37 °C in the lower right part of this axis, whereas Icatu plants showed a wider variation. However, it seems remarkable that at the highest temperature, the CO_2_ condition become determinant with PC1 separating the plants under aCO_2_ (left upper quadrant) from those under eCO_2_ (right upper quadrant).

### 2.3. Differential Gene Expression Changes in Response to Supra-Optimal Temperatures

**Figure 2:**
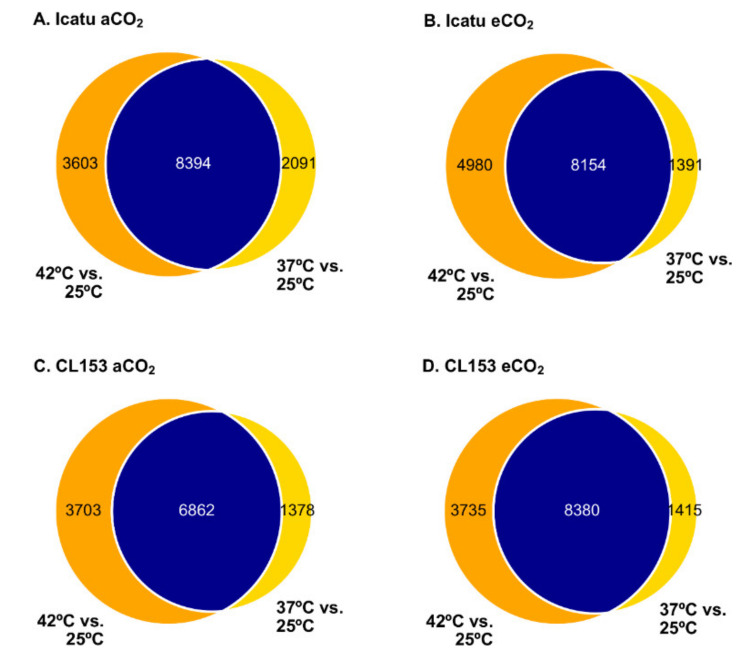
Transcriptional patterns among differentially expressed genes (DEGs) between the two supra-optimal temperatures of 37/28 °C (37 °C) and 42/30 °C (42 °C). Numbers represent the DEGs shared between treatments (blue), specific for 37 °C (yellow) and specific for 42 °C (orange), found in plants of Icatu (**A**,**B**) and CL153 (**C**,**D**) grown under 380 μL L^−1^ aCO_2_ or 700 μL L^−1^ eCO_2_.

An average of 11,170 and 11,833 DEGs were identified, respectively by DESeq2 and edgeR, with 93.34% identified by both tools. Detailed results of each method are depicted in Table S2. The number of DEGs varied from 9545 (37 °C; eCO_2_) to 13,134 (42 °C; eCO_2_) in Icatu and from 8240 (37 °C; aCO_2_) to 12,115 (42 °C; eCO_2_) in CL153, being consistently higher at the highest temperature in both species, especially when combined with eCO_2_ (Figure 2). Under the same CO_2_, the majority of DEGs were shared between the two supra-optimal temperatures (Figure 2).

In Icatu, 14.6% (eCO_2_) to 19.9% (aCO_2_) of DEGs were specific of 37 °C, while 30.0% (aCO_2_) to 37.9% (eCO_2_) were specific of 42 °C (Figure 2). As such, a higher number of treatment-specific DEGs were reported with the rise of temperature, especially under eCO_2_ where the highest number of DEGs were found (4980). This reflects a very specific response to the highest temperature that was further amplified by eCO_2_ (Figure 2A,B).

In comparison, CL153 showed a similar global % of specific DEGs with 14.4% to 16.7% of DEGs being expressed at 37 °C, and 30.8% to 35.0% DEGs at 42 °C (Figure 2), in eCO_2_ and aCO_2_ plants, respectively. However, although the absolute value of treatment-specific DEGs in CL153 was similar under aCO_2_ (3703) and eCO_2_ (3735), as temperatures rose its percentage was lower under eCO_2_ than under aCO_2_ (31% vs. 35%) (Figure 2C,D).

**Figure 3:**
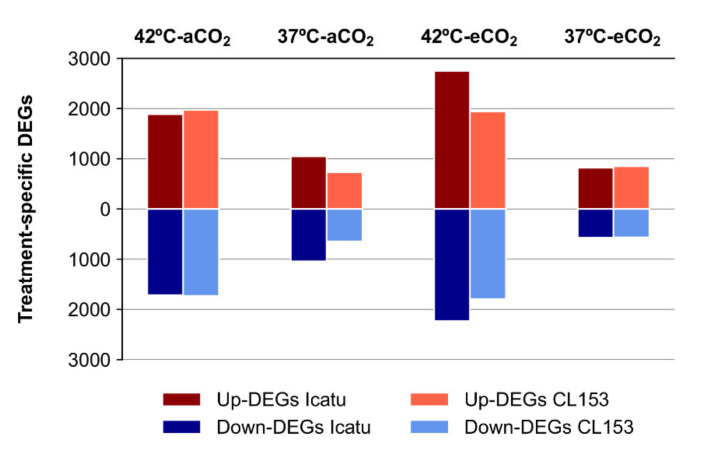
The effect of the supra-optimal temperatures 37/28 °C (37 °C) and 42/30 °C (42 °C) on the number of up- and down-regulated treatment-specific DEGs in plants of Icatu and CL153, grown in either 380 μL L^−1^ aCO_2_ or 700 μL L^−1^ eCO_2_.

**Figure 4:**
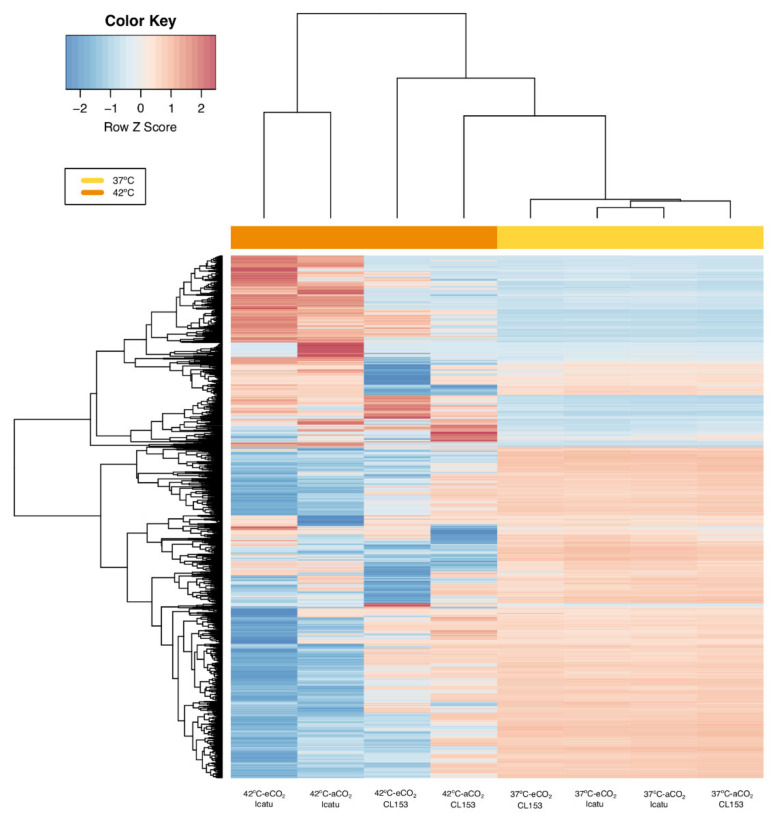
Clustered heat maps and dendrograms of the normalized log_2_ fold change visualizing the expression of treatment-specific differentially expressed genes (DEGs) in Icatu and CL153 as a response to 37/28 °C (37 °C) and 42/30 °C (42 °C) temperatures under 380 μL L^−1^ aCO_2_ or 700 μL L^−1^ eCO_2_. Significant DEGs were filtered by log_2_ fold change (FC) > |2| and the plotted values scaled by row using Z-scores. Hot colors represent up-regulated DEGs and cold colors represent down-regulated DEGs. Column color labels group comparisons by temperature treatments (yellow: 37 °C; orange: 42 °C).

The number of up- and down-regulated DEGs in plants at 37 °C and 42 °C was nearly the same in both genotypes, except at 42 °C in eCO_2_ where DEGs were predominantly up-regulated in Icatu (Figure 3). The expression profiles of specific DEGs were particularly influenced by the highest temperature, especially in Icatu which displayed a higher degree of variation (Figure 4). By contrast, the expression profile of the two genotypes exhibited a small differentiation at 37 °C.

### 2.4. Significantly Enriched GO Terms of Responsive DEGs in the Two Genotypes

In both species, an average of 74% of DEGs were annotated with gene ontology (GO) terms using the functional annotation of the reference genome of *C. canephora* (Table S3). Temperature increase had a negative effect on enriched GO terms, especially under eCO_2_.

**Figure 5:**
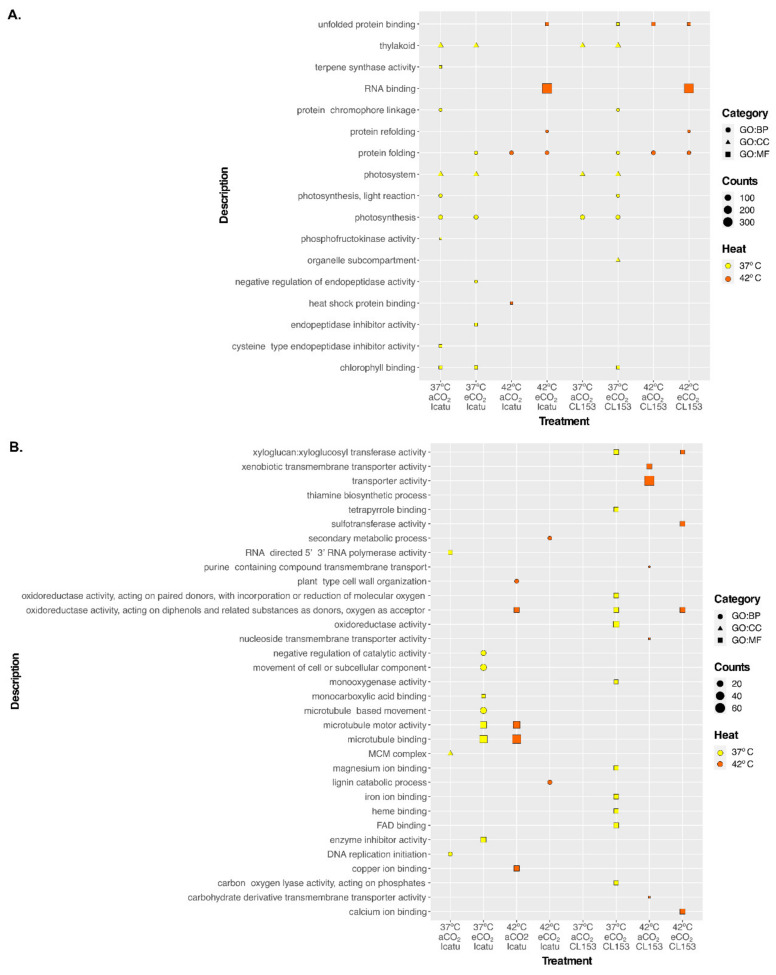
Over-representation analysis of gene ontology (GO) terms performed with gProfiler against the functional annotation of the *Coffea canephora* genome. Significantly (false discovery rate (FDR) < 0.01) enriched gene ontology (GO) terms among up-regulated (**A**) and down-regulated (**B**) differentially expressed genes (DEGs) in Icatu and CL153 were ranked by increasing log_2_ fold change (FC), considering the effect of supra-optimal temperatures at 37/28 °C (37 °C), and 42/30 °C (42 °C) in plants grown in 380 μL L^−1^ aCO_2_ or 700 μL L^−1^ eCO_2_. The absence of a treatment in the figure indicates that no enriched GO terms were found. GO terms are grouped by the main categories: biological process (GO:BP), molecular function (GO:MF), and cellular component (GO:CC). Counts indicate the number of DEGs annotated with each GO term and dots are colored by temperature treatment.

Up-regulated DEGs at 37 °C showed more enriched GO terms than at 42 °C, under both CO_2_ conditions (Figure 5A; Table S4). Within the same temperature treatment, eCO_2_ triggered more enriched GO terms than aCO_2_. The most enriched GO term (almost 300 genes) was related to molecular functions (RNA binding, GO:0003723) and was found only at 42 °C in eCO_2_. The remaining were less enriched categories with a predominance of GO terms related to photosynthesis (GO:0015979), thylakoid (GO:0009579), photosystem (GO:0009521), photosynthesis, light reaction (GO:0019684), and chlorophyll binding (GO:0016168) found only at 37 °C. Up-regulated DEGs in 42 °C Icatu plants were also enriched for heat shock protein binding (GO:0031072) but only under aCO_2_. In fact, at 42 °C in eCO_2_ there was an enrichment in the folding and binding of proteins.

Down-regulated DEGs featured 37 enriched categories (17 in Icatu and 20 in CL153), although all with a very small number of gene counts (Figure 5B; Table S5). At 37 °C, down-regulated DEGs showed almost no enriched categories under aCO_2_ while under eCO_2_, Icatu and especially CL153 showed an enrichment in general activities as for instance, oxidoreductase activity acting on paired donors with incorporation or reduction of molecular oxygen (GO:0016705). At 42 °C in aCO_2_, down-regulated DEGs linked with microtubule motor activity (GO:0003777) and microtubule binding (GO:0008017) were highly enriched in Icatu while CL153 showed an enrichment in the molecular functions related to transporter activity. In this supra-optimal temperature, eCO_2_ triggered less enriched categories: Icatu plants were mostly enriched in two biological processes, the secondary metabolic process (GO:0019748), and the lignin catabolic process (GO:0046274), while CL153 plants showed an enrichment in the molecular functions linked to calcium ion binding (GO:0005509), xyloglucan:xyloglucosyl transferase activity (GO:0016762), sulfotransferase activity (GO:0008146), and oxidoreductase activity, acting on diphenols and related substances as donors, oxygen as an acceptor (GO:0016682).

### 2.5. Effect of Supra-Optimal Temperatures and eCO_2_ on Photosynthetic and Other Biochemical-Related Responsive DEGs

A total of 667 responsive DEGs associated with photosynthesis and other important metabolic functions in coffee leaves (e.g., antioxidant mechanisms, lipids, and respiratory pathways) were found in the two genotypes: 180 directly related with the photosynthetic pathway (122 to photosynthesis, 49 to the chlorophyll metabolic process, 9 to RuBisCO activity), 73 to antioxidant activities, 123 to lipid metabolism, and 291 directly related with the respiratory pathway (170 to cellular respiration, 30 to malate dehydrogenase activity, and 91 to pyruvate kinase activity) (Table S6). Fold changes of these DEGs varied widely but the most extreme values were always found at 42 °C.

**Figure 6:**
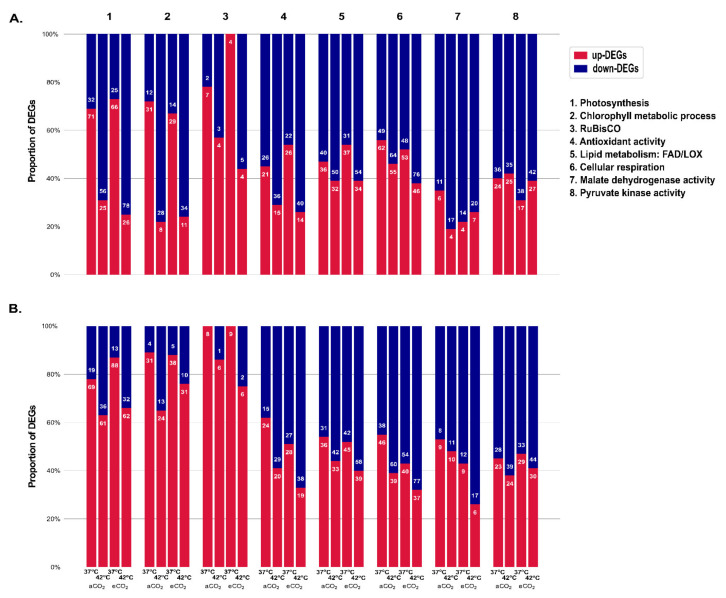
Changes in the proportion of the regulation (%) and in the numbers (indicated in each bar) of differentially expressed genes (DEGs) related to photosynthesis and biochemical processes in Icatu (**A**) and CL153 (**B**) plants, as a response to 37/28 °C (37 °C) and 42/30 °C (42 °C) and grown in 380 μL L^−1^ aCO_2_ or 700 μL L^−1^ eCO_2_. The searched gene ontology (GO) biological processes included: “photosynthesis”, “chlorophyll metabolic process”, “ribulose-bisphosphate carboxylase activity” (RuBisCO), “antioxidant activity”, “lipid metabolic process (LOX, FAD)”, “cellular respiration”, “malate dehydrogenase activity”, and “pyruvate kinase activity”, as well as its direct child significant terms.

**Figure 7:**
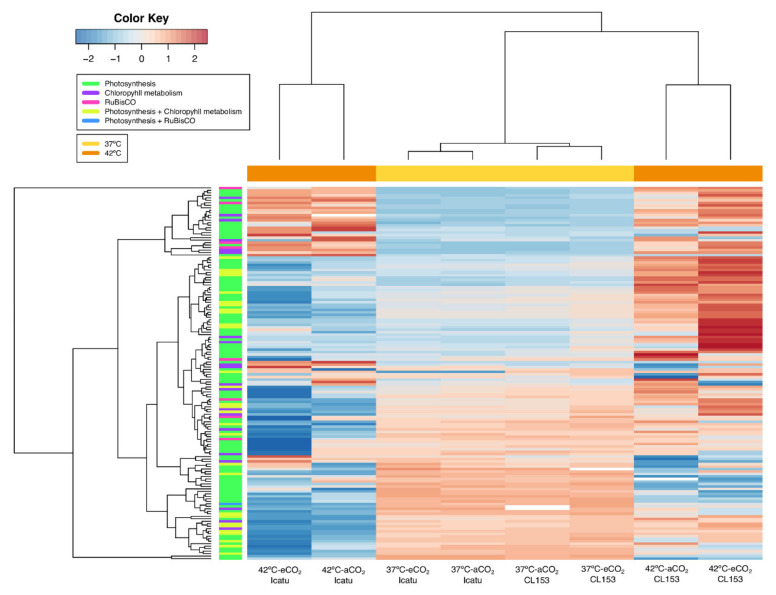
Heatmap and dendrograms of the normalized log_2_ fold change (FC) of photosynthesis-related significant differentially expressed genes (DEGs) of Icatu and CL153 plants as a response to 37/28 °C (37 °C) and 42/30 °C (42 °C) and grown in 380 μL L^−1^ aCO_2_ or 700 μL L^−1^ eCO_2_. DEGs presented here are annotated with the gene ontology (GO) terms “photosynthesis”, “chlorophyll metabolism process”, and “RuBisCO”, according to the functional annotation of the *Coffea canephora* genome. The plotted values were scaled by row for improved visualization. Hot colors represent up-regulated DEGs, and cold colors represent down-regulated DEGs. Column color labels group comparisons by temperature treatment, while row color labels group genes by GO annotation.

Overall, a similar number of photosynthetic DEGs were found in the two genotypes as a response to supra-optimal temperatures although with contrasting levels of expression. These DEGs were mostly up-regulated at 37 °C but down-regulated at 42 °C in Icatu (Figure 6A), while the majority were up-regulated in CL153 (Figure 6B and Figure 7). The same pattern was found for DEGs involved in the chlorophyll metabolic process (Figure 6 and Figure 7). In comparison, DEGs linked to RuBisCO activity were mostly down-regulated in Icatu plants under 42 °C independently of CO_2_, while in CL153 they were always up-regulated (Figure 6 and Figure 7). More than half of the photosynthesis DEGs were involved in binding activities (Table S6). Notably, under 42 °C and independently of the CO_2_ conditions, DEGs related to the reaction centres of photosystems (PSs) I and II were down-regulated in Icatu, contrary to CL153 where they were up-regulated. Under these extreme conditions, Icatu also repressed genes involved in chlorophyll a-b binding and most PsbQ and PsbP genes, contrary to CL153 where they were generally up-regulated (Table S6).

The remaining categories were mostly down-regulated, especially at 42 °C under eCO_2_ although differences were still found between the two genotypes (Figure 6A,B). For instance, DEGs involved in antioxidant activities and lipid metabolism were always more down-regulated in Icatu except at 37 °C in eCO_2_, while in CL153 those DEGs were always up-regulated at 37 °C, independently of CO_2_ (Figure 6A,B). Overall, in Icatu plants under both CO_2_ conditions, the number of up-regulated DEGs involved in cellular respiration was higher in 37 °C plants than in the 42 °C plants. In CL153, a high number of up-regulated DEGs were found, but only in 37 °C plants under aCO_2_. DEGs involved in pyruvate kinase (PK) and malate dehydrogenase (MDH) activity (involved in glycolysis) were mostly down-regulated in all treatments, except for MDH in 37 °C-CL153 plants under aCO_2_.

## 3. Discussion

### 3.1. Unveiling the Transcriptomic Responses of Coffee to Supra-Optimal Temperatures and eCO_2_

High temperatures cause large morphological, physiological, and biochemical effects limiting plant growth and productivity [44,45]. They also have a major impact on plant transcriptomes resulting in a high number of DEGs when compared to other stresses, although the extent and composition vary widely between species [46]. In this study, we found that supra-optimal temperatures triggered specific transcriptomic changes in two coffee genotypes, belonging to different species. Under the same temperature conditions, the polyploid *C. arabica* cv. Icatu expressed more genes than the diploid *C. canephora* cv. Conilon Clone 153. However, both showed a decrease in the number of responsive genes to the rising temperatures, especially under the highest one (42 °C) (Figure 1A). A marked effect was clearly reflected in the distribution of the expressed genes plotted in the PCoA, where plants at 42 °C differed among CO_2_ conditions and were well separated from plants at 25 °C and 37 °C (Figure 1B). Samples exposed to 25 °C and 37 °C clustered closely in the PCoA supporting previous physiological and biochemical findings showing that these genotypes did not suffer significant negative impacts up to 37 °C (compared to their controls at 25 °C), regardless of the CO_2_ level, although with a better performance under eCO_2_, especially as regards the photosynthetic pathway [14,30,41].

The number of DEGs specific to each supra-optimal temperature was very high, and higher at 42 °C than at 37 °C (Figure 2 and Figure 3). A clear differentiation of genes was detected at 42 °C (Figure 4) supporting the strongest influence of the highest temperature in the number of DEGs found in the two species. These DEGs are probably critical for enhancing temperature tolerance in Icatu and CL153, in contrast with other varieties of *C. arabica* (cv. Acauã and Catuaí) where the number of DEGs decreased as a response to elevated temperatures (23/19 °C vs. 30/26 °C [35]). Nevertheless, these differences might also be due to the short period of the experimental trial (e.g., 4 weeks [35] vs. 10 months in our present study), and to the physiological performance of coffee plants which is frequently higher at 30 °C than at 25 °C [14,30].

Despite the influence of the temperature, the majority of DEGs were commonly expressed in both supra-optimal temperatures, since only 14.6% to 37.9% of DEGs in Icatu, and 14.4% to 35.0% of DEGs in CL153, were specific to 37 °C or 42 °C (Figure 2). Globally, DEGs had a similar level of regulation in the two species except at 42 °C under eCO_2_, where a higher up-regulation was seen in Icatu than in CL153 (Figure 4). Thus, supra-optimal temperatures triggered a common transcriptomic response in the two coffee genotypes, so that conserved responsive genes were activated, which is highly likely to occur due to the close genetic relationship of the two genotypes. Genome assembly of the allotetraploid *C. arabica* supports a high level of genetic sharing with the diploid progenitors, *C. canephora* and *C. eugenioides* [20]. Comparative studies between coffee species and genotypes are scarce, but the few existing ones are in line with our findings and suggest some common responses to supra-optimal temperatures [36,41,47] and a bottleneck effect in transcriptional activation at warmer temperatures [35]. On the other hand, eCO_2_ increased the numbers at 42 °C in both genotypes (Figure 2). However, the increase of specific DEGs triggered at 42 °C in eCO_2_, especially those seen in Icatu, shows a specific response to this extreme temperature, which could be related to a higher temperature resilience and crop acclimation capability of these plants under eCO_2_ than under aCO_2_ [14,40,41].

Supra-optimal temperatures showed an enrichment in the up-regulation of RNA binding at 42 °C, both in Icatu and CL153 (Figure 6). This is consistent with the findings in rice [48], wheat [49], and maize [50] suggesting that temperature stress has a general conserved response across plant lineages, where binding and folding play an important role in the ability of a plant to cope with stress. At 37 °C we also found an enrichment in the up-regulation of thylakoid, photosystem, and photosynthesis in both species and under CO_2_ conditions (Figure 5A), in line with previous results demonstrating the intrinsic ability of these plants to maintain the photosynthetic activity up to 37 °C, regardless of air CO_2_ but with a clear negative impact at 42 °C [14,30]. This would explain why these categories are no longer enriched at that temperature. Nonetheless, eCO_2_ seem to mitigate some of the impacts imposed by 42 °C at least in Icatu, since heat-shock proteins, which are usually up-regulated during stress events, were only enriched under aCO_2_ (Figure 5A).

Little attention has been given to the decrease of transcripts during stress imposition, but our results suggest that decreases in specific transcripts might play an important role in plant acclimation. A strong difference was found in the response to the studied temperatures, since gene enrichment in down-regulated DEGs was mostly felt at 37 °C than at 42 °C, in both species (Figure 5B). Previous reports showed that these genotypes retain the ability to cope with temperatures as high of 37 °C (or even 39 °C), but at 42 °C the tolerance capability is overcome [14,30]. In fact, at 37 °C, down-regulated DEGs showed almost no enriched categories under aCO_2_ while under eCO_2_, Icatu and especially CL153 showed an enrichment in general activities as oxidoreductase activity acting on paired donors with the incorporation or reduction of molecular oxygen (GO:0016705). An important point of our experiments is that coffee plants were gradually exposed to supra-optimal temperatures, allowing them to express their acclimation capabilities under 37 °C (and partly 39 °C). This intrinsic capability to acquire thermotolerance would contribute to metabolic homeostasis, even under fluctuating field environments, explaining why these genotypes perform better under elevated temperatures than expected in classical reports [36]. Nevertheless, these reports also showed that eCO_2_ allowed the plant to maintain a greater metabolic activity than under aCO_2_, particularly at 42 °C. In fact, at that temperature, we found less enriched categories in down-regulated DEGs under eCO_2_ than aCO_2_. At 42 °C in aCO_2_, down-regulated DEGs in Icatu were enriched in microtubule motor activity (GO:0003777) and microtubule binding (GO:0008017), while in CL153 they showed an enrichment in transporter activity, being possibility involved in the response to stress. Although mechanisms are still being studied, several studies suggest that microtubules act as sensors of abiotic stressors, changing cellular transport, division, and the formation of the cell wall in response to stresses such as drought and temperature [51]. In addition, some major tolerance mechanisms include transporter proteins that are involved in signaling cascades [52]. In this supra-optimal temperature, eCO_2_ seem to alleviate some stress effects and only six categories were enriched (Figure 5B). In down-regulated DEGs, *C. arabica* showed an enrichment in the secondary metabolic and lignin catabolic processes, while *C. canephora* mainly enriched molecular functions linked to calcium ion binding, oxidoreductase, sulfotransferase, and xyloglucan:xyloglucosyl transferase activity, suggesting a decrease in the production of lignin in *C. arabica* and of cellulose in *C. canephora*. Xyloglucan:xyloglucosyl transferases constitute cell wall-modifying enzymes that play a fundamental role in the cell wall expansion and re-modelling [53]. In addition, cellular Ca^2+^ levels have a direct link with the expression of genes involved in stress responses having, in some cases, a negative function in regulation. For instance, plants overexpressing the nuclear CaMBP25 binding protein show increased sensitivity to salt and osmotic stresses while antisense lines were found to be more tolerant [54]. Sulfotransferases have a broad range of substrates, but some studies also indicate functions in stress response and plant development [55].

### 3.2. Transcriptomic Impacts of Supra-Optimal Temperatures and eCO_2_ on Photosynthesis and Biochemical-Related Processes

The activation of defense processes often negatively affects the growth of plants [56,57], and although this tradeoff is fundamental for survival in changing environmental conditions it also affects plant yield [58]. In the next half century, a significant change in agricultural production is needed to assure food security of the growing human population, while coping with a changing climate [59,60]. In nature, the presence of multiple environmental changes is common, and as such, there has been an increase in the number of studies addressing this topic, which often show synergistic to antagonist plant responses when compared to single environmental changes [61,62]. For instance, the eCO_2_ “fertilization” effect when combined with elevated temperatures, increases the thermal optimum for carbon fixation [63]. Although high temperatures and eCO_2_ interact in ways that can either exacerbate or diminish their independent effects [64], several reports have highlighted that eCO_2_ can mitigate the negative impacts of elevated temperatures. It increases tree growth and forest productivity [65], and the photosynthetic tolerance in some C3 plants, including of the coffee genotypes studied here [14,36,41]. We have previously shown that under the single factor of eCO_2_, CL153 mostly expressed DEGs associated with general biological processes and to a lower extent to abiotic stress responses, while Icatu increased the upregulation of genes linked to plant tolerance and adaptation to abiotic factors [43]. The positive effects of eCO_2_ in CL153 were also related to an enrichment in the cutin, suberine, and wax KEGG pathway in CL153, which would help to generate more biomass and height than under aCO_2_. GO terms associated with RuBisCO were found to be largely up-regulated in Icatu and in CL153 under the single exposure to eCO_2_, supporting the absence of photosynthetic acclimation and the reinforcement of RuBisCO activity [13,14]. In addition, a specific search of the processes that play a major role on the physiological and biochemical acclimation of coffee to several environmental constraints resulted in the identification of 171 DEGs in Icatu and 190 DEGs in CL153 that were significantly affected by eCO_2_ [43], with these numbers being much lower than the ones found here for the combined effects of eCO_2_ and high temperatures.

In the present study, 667 DEGs associated with photosynthetic and other biochemically important metabolic functions were affected by supra-optimal temperatures and eCO_2_. As compared to 25 °C, in Icatu, DEGs involved in the photosynthesis and the chlorophyll metabolic processes were mostly up-regulated at 37 °C but down-regulated at 42 °C, while in CL153 they were up-regulated in both supra-optimal temperatures. This suggests a higher impact in the photosynthetic apparatus at 42 °C in Icatu under aCO_2_, which also showed a down-regulation of DEGs involved in the PSs I and II reaction centers, in contrast to CL153 where they were up-regulated. However, it has been shown that the PS activity was only moderately affected at 42 °C in both species, and to an extent barely higher in Icatu than in CL153, as compared to their maximal activity at 37 °C, under aCO_2_, although the performance was not that far from the one measured at 25 °C [14]. In fact, although PSs can be temperature sensitive sites [9] altering electron transport [66], these coffee genotypes have a high tolerance in the components of their photosynthetic machinery until 37 °C, being moderately affected at 42 °C [14,30]. Additionally, eCO_2_ relieves the impact of the 42 °C exposure as regards the global functioning of the photosynthetic apparatus (reflected in the photosynthetic capacity, A_max_), particularly at a PSs level (e.g., in the maximal, F_v_/F_m_, and actual F_v_’/F_m_’, photochemical efficiency of PS II, and the estimate of quantum yield of photosynthetic non-cyclic electron transport, Y_(II)_) [14]. Therefore, the large down-regulation of DEGs at 42 °C found here for photosynthesis (irrespective of air CO_2_), especially in Icatu, when compared with the numbers at 37 °C (Figure 6), suggests that CL153 and Icatu support their (partial) resilience at 42 °C through protective mechanisms (e.g., energy dissipation molecules, electron flow around PS I, HSP70) of the existing thylakoid photochemical components [30,41] rather than through de novo synthesis for the substitution of damaged structures.

On the other hand, the RuBisCO activity increases continuously up to 37 °C in Icatu and CL153, followed by a sharp negative impact at 42 °C, being the most temperature sensitive (biochemical) component of the photosynthetic apparatus in contrast with (photochemical) PS components [14,30], and without any mitigation effect of eCO_2_ [14]. These earlier findings are in line with the high number of up-regulated DEGs associated with RuBisCO found here at 37/28 °C, especially in CL153 where a higher enzyme activity was shown, particularly under eCO_2_ [14]. Therefore, such a high number of up-regulated DEGs (and the increase in RuBisCO content thereafter) is likely crucial to the maintenance of the increased photosynthetic activity at 37 °C, under both air CO_2_ conditions (although higher under eCO_2_) [14,30]. The strong down-regulation of DEGs related with RuBisCO at 42 °C, including RuBisCO activase (Table S6) that is known to be particularly sensible to stress, are also congruent with the observed decrease in RuBisCO activity [14,30], especially in Icatu under eCO_2_, which had the lowest number of up-regulated DEGs at 42 °C (Figure 6) together with the lowest total activity of RuBisCO [14]. Altogether, these results suggest a close association between RuBisCO activity and the regulation of their related DEGs that can be used as key probes to evaluate the temperature resilience of coffee plants.

Several genes coding for the photosynthetic machinery (*PsbQ* and *PsbP*) exhibited a reduced expression in Icatu in response to supra-optimal temperatures contrary to CL153 where they were generally up-regulated (Table S6). These auxiliary proteins, namely those that are *PsbP*-like, are required for the assembly, stability, and repair of PS II [67,68], and their down-regulation in Icatu suggests a higher impact of supra-optimal temperatures in this genotype than in CL153, as reflected in the lower F_v_/F_m_ under aCO_2_ in Icatu [14]. Other studies have reported few differences in relation to the genes linked to photosynthesis between the allopolyploid *C. arabica* (cv. Caturra) and its progenitors (*C. canephora* cv. Nemaya and *C. eugenioides*) when grown under a constant temperature of 24 °C [47]. This has been explained by a higher adaptive ability of the polyploid in comparison to the diploid progenitors [69], although more studies are needed to understand the key effects of extreme temperatures. In our case, it should be highlighted that an extensive physiological and biochemical analysis of the same plants used in this work showed minor impacts (if at all) at 37 °C in most photosynthetic-related parameters, including net and maximal photosynthetic rates, PS II photochemical efficiency, thylakoid electron transport and carriers, and the activity of enzymes (including RuBisCO), regardless of air CO_2_ [14]. However, at 42 °C, a damage threshold was clearly reached for these two genotypes considering most photosynthetic-related parameters [14,30]. As referred, photosynthetic biochemical components, represented by RuBisCO and Ru5PK enzymes (similarly to the NADH-dependent malate dehydrogenase, and pyruvate kinase, involved in the respiratory pathway) were by far the most sensitive components of these genotypes at 42 °C.

Several antioxidants and lipids have been described as common and determinant components in the acclimation response of coffee plants to a wide range of environmental stresses, including high irradiance [70], cold [17,71,72], and temperature [41,73]. In fact, at the chloroplast level, both antioxidative enzymes and the strong lipid dynamics of chloroplast membranes, with qualitative modifications in the fatty acid unsaturation and in the balance of lipid classes (mostly in Icatu), seem to contribute to support photosynthetic functioning at 37 °C independently of CO_2_, and at 42 °C only under eCO_2_ [41,73]. In accordance, in this study, we found that DEGs related to both antioxidant activity and lipid metabolism (FAD/LOX) showed a higher up-regulation at 37 °C than at 42 °C, regardless of air CO_2_ and genotype, further reflecting the difficulties of these plants in enduring the most extreme temperature (42 °C). Several antioxidant genes such as peroxidase 4, or thioredoxin and FAD-related genes such as Reticuline oxidase (Table S6), might be useful probes to evaluate the ability of temperature acclimation in coffee genotypes.

In summary, an intrinsic temperature resilience was observed in both Icatu and CL153 until 37 °C, irrespective of the air CO_2_, although with a global higher photosynthetic performance under eCO_2_. At 42 °C, clear negative impacts were observed, namely in RuBisCO, PSs, and the thylakoid electron carriers for both CO_2_ conditions, but globally these were more severe under aCO_2_ and for Icatu in comparison with CL153 [14,30]. Nevertheless, these studies do not fully agree with the variation of transcript profiles in the present work suggesting the presence of post-transcriptional changes that modify the relation between transcription and the biochemical result, showing the importance of placing transcript profiling results in the context of physiological and biochemical complementary studies.

### 3.3. Different Transcriptomic Responses: Potential Implications for the Coffee Industry

Coffee had a transformative role in our history, especially after the 20th century when most varieties were created to meet an increasing coffee consumption demand [19]. However, coffee breeding is largely restricted to two species—the polyploid *C. arabica* and one of its ancestors, the diploid *C. canephora*, which together dominate the world production [15]. Both are highly affected by climate change [74]. A major task is therefore to create the next generation of coffee cultivars adapted to the future climate conditions, by unlocking resilience traits while maintaining global productivity and the high quality of coffee beans. Until now, *C. canephora* has provided a major source of desirable traits to *C. arabica* cultivars, including those associated with disease and pest resistance, such as the coffee berry disease (*Colletotrichum kahawae* [75]) that was not inherent in *C. arabica*. Predicted global warming might endanger this crop sustainability in several producing regions [76,77], while increasing the extinction risk for wild coffee species [78], despite findings that eCO_2_ can preserve plant physiological performance and mitigate temperature and drought impacts [14,38,40], enhancing crop yield [36] and bean quality [42]. Therefore, studies in coffee genotypes that outperform others when exposed to higher temperatures are a priority in coffee breeding programs [29,37] to guarantee the sustainability of the entire chain of value for this tropical agricultural product consumed worldwide.

In this study, we provide new clues as how *C. arabica* and *C. canephora* genotypes can acclimate to projected future climate changes by exploring the effects of elevated temperatures alone, and in combination with eCO_2_. Both Icatu and CL153 revealed an intrinsic tolerance until 37 °C, which is well above what is usually assumed, but both were clearly impacted at 42 °C [14,30]. Changes in transcriptomic profiles suggested that the diploid CL153 might be less affected under aCO_2_ by the extreme temperature of 42 °C than the polyploid Icatu, although both genotypes presented severe impacts in the leaf metabolism including the photosynthetic level [14,30,41]. Nevertheless, mitigation by eCO_2_ of the negative impacts imposed by this high temperature was not entirely reflected in the up-regulation of DEGs associated with photosynthetic genes (particularly in Icatu), suggesting that the maintenance of photosynthetic activity was preferentially supported by protective mechanisms rather than de novo protein synthesis. Still, the identification of key genes related with photosynthesis can provide clues to the temperature tolerance/sensitivity of coffee genotypes. Taken together with previous physiological and biochemical studies, this RNAseq study provides evidence of acclimation responses that underpin key processes for coffee acclimation to extreme temperatures.

## 4. Materials and Methods

### 4.1. Plant Material and Experimental Design

In this study, we used 1.5 years old potted plants from two cultivated Brazilian genotypes, *C. canephora* Pierre ex A. Froehner cv. Conilon Clone 153 (CL153) and *C. arabica* L. cv. Icatu Vermelho (Icatu); the latter an introgressed variety resulting from a cross of *C. canephora* and *C. arabica* cv. Bourbon Vermelho, then further crossed with *C. arabica* cv. Mundo Novo). The experimental conditions were implemented as previously described [29]. Briefly, plants were grown in 28 L pots in walk-in growth chambers (EHHF 10000, ARALAB, Portugal) during 10 months under controlled environmental conditions of temperature (25/20 °C, day/night), relative humidity (ca. 70%), irradiance (ca. 700–800 μL L^−1^), photoperiod (12 h), and either at ambient (aCO_2_: 380 μL L^−1^) or elevated (eCO_2_: 700 μL L^−1^) CO_2_. Thereafter, temperature was raised at a rate of 0.5 °C day^−1^ (diurnal temperature) from 25/20 °C to 42/30 °C, with 7 days of stabilization at 31/25, 37/28, and 42/30 °C to allow for programmed evaluations [14,41]. The impacts of increased temperatures were studied for a moderate supra-optimal temperature (37 °C; 37/28 °C), and an extreme supra-optimal temperature (42 °C; 42/30 °C), in comparison to the control temperature (25 °C; 25/20 °C), always at aCO_2_ or eCO_2_ conditions. The value for the eCO_2_ treatment was chosen considering the predictions for the second half of the current century [2]. The plants were grown in an optimized substrate consisting of a mixture of soil, peat, and sand (3:1:3, v/v/v) [79] and fertilized as previously described [13].

### 4.2. RNA Extraction, Illumina Sequencing, and Data Quality Control

Leaves from plagiotropic and orthotropic branches were collected after ca. 2–3 h of illumination (between 10:00–11:00) from the two top pairs of recently matured leaves in the upper (illuminated) part of each plant (nine per treatment), flash frozen in liquid nitrogen and stored at −80 °C until analysis. Total RNA was extracted from 36 samples (two genotypes × two CO_2_ treatments × three temperatures × three biological replicates) using the RNeasy Plant Mini Kit (Qiagen, Cologne, Germany) according to the manufacturer’s instructions. All RNA samples were individually analyzed for the possible presence of DNA contamination by standard PCR reactions (35 cycles) using primers designed for ubiquitin (UBQ) gene, in the absence of cDNA synthesis [71]. Quantity and quality of the RNA was determined using a BioDrop Cuvette (BioDrop, Cambridge, UK) and an Agilent 2100 Bioanalyzer (Agilent Technologies, Santa Clara, CA, USA). The RNA integrity number (RIN) for the samples ranged from 8.20 to 9.20. The messenger RNA (mRNA) libraries were constructed with the Illumina “TruSeq Stranded mRNA Sample Preparation kit” (Illumina, San Diego, CA, USA) and sequenced separately on Illumina Hiseq 2000 at the MGX platform (Montpellier GenomiX, Montpellier, France) using 1 × 50 bp single-end reads at a depth of 1.4 GB.

High-quality reads were obtained after several steps of quality checks which included trimming and the removal of adaptor/primer and low-quality reads using FastQC version 0.11.8 [80] and Trimmomatic version 0.38 [81] through the trimming steps: ILLUMINACLIP to cut adaptors, SLIDINGWINDOW:4:15 to trim low-quality reads, and MINLEN:38 to drop small reads. FastQ Screen version 0.13 [82] was used to check for contaminants against the genome of the most common model organisms (e.g., *Homo sapiens*, *Mus musculus*, *Rattus norvegicus*, *Drosophila melanogaster*, *Caenorhabditis elegans*, *Saccharomyces cerevisiae*, and *Escherichia coli*) and adapter databases (e.g., mitochondria RNA, PhiX, Vector from UniVec database, FastQ Screen rRNA custom database, and FastQ Screen Adapters database).

### 4.3. Reference-Based Mapping and Assembly

The filtered high-quality reads were mapped to the reference genome of the *C. canephora* genome downloaded from the Coffee Genome Hub (http://coffee-genome.org/download, accessed on 13 July 2019) [83] using STAR version 2.6.1 [84] with default settings, including quantMode set to GeneCounts. Htseq-count v0.11.0 [85] was used with the default mode “union” and the option “stranded = reverse” to count only uniquely mapped reads to each gene, and discarding reads in multiple alignments to avoide the increase of false positives. Samtools version 1.9 [86] and gffread version 0.9.9 [87] were used throughout the analysis to obtain general statistics of genome mapping.

### 4.4. Identification of Differentially Expressed Genes (DEGs)

Gene expression normalization of all the samples was estimated in FPKM (fragments per kilobase of exon per million fragments mapped). The changes in the relative abundance of the genes between the different genotypes/CO_2_ treatments/temperature treatments were estimated using DESeq2 v1.28.1 [88] and edgeR v3.30.3 [89]. Only the DEGs identified by both tools as significant were used in subsequent analyses. The resulting values were adjusted using the Benjamini and Hochberg’s approach for controlling the false discovery rate (FDR; [90]). Genes with a normalized non-zero log_2_ fold change expression and an FDR < 0.01 were defined as differentially expressed. Python’s matplotlib library was used to plot Venn diagrams and barplots [91]. Principal coordinate analysis (PCoA) was performed on the expression data of genes, FKPM normalized and log10-transformed, using the function prcomp in R software version 3.5.1 [92].

### 4.5. Functional Classification of Response DEGs

DEGs followed the functional annotation of the reference genome *C. canephora*, downloaded from the Coffee Genome as described above. Gene ontology (GO) enrichment analyses were applied to understand the functional classification of responsive DEGs through an over-representation analysis (ORA) using gProfiler [93] under FDR < 0.01. Results were summarized using REVIGO [94] by removing redundant GO terms with an allowed similarity = 0.5. Enrichment non-redundant results were plotted using the R ggplot2 version 3.3.2 library [95]. This same package was used to plot heatmaps with dendrograms to visualize DEGs based on the differential expression patterns found between the different treatments. To prevent highly differentially expressed genes from clustering together without considering their expression pattern, the log_2_ fold change was scaled by gene across treatments (row Z-score).

## Data Availability

Raw reads have been deposited in the NCBI Sequence Read Archive, BioProject accession PRJNA630692.

## Supplementary Materials

The following are available online at https://www.mdpi.com/1422-0067/22/6/3125/s1.
