# Supplementary material for: A Transcriptomic Approach to Understanding the Combined Impacts of Supra-Optimal Temperatures and CO2 Revealed Different Responses in the Polyploid Coffea arabica and Its Diploid Progenitor C. canephora
Source: Int J Mol Sci. 2021 Mar 18;22(6):3125. doi: 10.3390/ijms22063125 (PMC8003141; doi:10.3390/ijms22063125)
Supplement: Supplementary file 1 [file ijms-22-03125-s001.pdf]

SUPPORTING INFORMATION TABLES

Table S1. Summary of sequencing data and mapped reads for the samples of *Coffee arabica* cv. Icatu (Icatu) and *C. canephora* cv. CL153 (CL153). A, B, and C correspond to biological replicates. Plants were submitted to control temperature at 25/20 °C (25 °C), 37/28 °C (37 °C), and 42/30 °C (42 °C), and grown in either 380 μL L^{-1} (aCO_2) or 700 μL L^{-1} (eCO_2). Raw reads: number of reads obtained after sequencing. Clean reads: number of reads passing the Illumina quality filters and downstream filters. % Clean: percentage of reads passing filters compared to the number of raw reads. Unique: number of reads aligned to a unique position. % Unique: proportion of reads aligned to a unique position compared to the number of clean reads. Multiple map: number of reads aligned to exons of several overlapping genes. % Multiple map: proportion of reads aligned to exons of several overlapping genes compared to the number of clean reads. % Unmapped: proportion of non-aligning reads compared to the number of clean reads.

GENOTYPE	[CO2]	TEMPERATURE	REPLICATES	RAW READS	CLEAN READS	% CLEAN	UNIQUE	% UNIQUE	MULTIPLE MAP	% MULTIPLE MAP	% UNMAPPED
**Icatu**	aCO_2	25ºC	1A	28702752	26442162	92.12	23198363	87.73	2397063	9.06	3.21
			1B	28603251	26372236	92.20	22181864	84.11	3371170	12.78	3.10
			1C	27795986	25107797	90.33	18468518	73.56	5832560	23.23	3.21
			Average	28367330	25974065	91.60	21282915	81.80	3866931	15.02	3.18
		37ºC	9A	28029335	26158146	93.32	22716484	86.84	2455191	9.39	3.77
			9B	26467175	24778725	93.62	21511856	86.82	2341940	9.45	3.74
			9C	26399997	24568206	93.06	21290969	86.66	2353694	9.58	3.76
			Average	26965502	25168359	93.34	21839770	86.77	2383608	9.47	3.75
		42ºC	2A	35347710	32590153	92.20	28115558	86.27	3265818	10.02	3.71
			2B	36899936	34008889	92.17	29893921	87.90	6249726	8.77	3.33
			2C	31079752	28583341	91.97	28583341	87.70	2567187	8.98	3.32
			Average	34442466	31727461	92.11	28864273	87.29	4027577	9.25	3.45
**Icatu**	eCO_2	25ºC	3A	30895839	29009249	93.89	24211896	83.46	3872399	13.35	3.19
			3B	25630485	23784221	92.80	18809838	79.09	4199675	17.66	3.26
			3C	31962251	29719153	92.98	23355288	78.59	5379009	18.10	3.31
			Average	29496192	27504208	93.22	22125674	80.38	4483694	16.37	3.25
		37ºC	10A	29875514	27911427	93.43	24177235	86.62	2678435	9.60	3.78
			10B	35512604	32842431	92.48	26519435	80.75	5117495	15.58	3.67
			10C	24494246	22612119	92.32	18398322	81.36	3397665	15.02	3.61
			Average	29960788	27788659	92.74	23031664	82.91	3731198	13.40	3.68
		42ºC	4A	25150070	23507568	93.47	18018450	76.65	4620766	19.66	3.70
			4B	26381269	24732239	93.75	19930547	80.59	3906648	15.80	3.62
			4C	23576551	22009513	93.35	16968954	77.10	4229150	19.21	3.68

			Average	25035963	23416440	93.52	18305983	78.11	4252188	18.22	3.66
**Average Icatu**				29044707	26929865	92.76	22575046	82.88	3790866	13.62	3.50
**CL153**	aCO_2	25ºC	5A	24532884	22853193	93.15	17904841	78.35	4300626	18.82	2.83
			5B	28922635	26926317	93.10	20799330	77.25	5356228	19.89	2.86
			5C	25702571	23940567	93.14	18619604	77.77	4675368	19.53	2.70
			Average	26386030	24573359	93.13	19107925	77.79	4777407	19.41	2.79
		37ºC	11A	28288493	25823242	91.29	20067870	77.71	4957015	19.20	3.10
			11B	27548373	25230205	91.59	19112734	75.75	5344280	21.19	3.07
			11C	27339032	25133850	91.93	20187078	80.32	4246226	16.89	2.79
			Average	27725299	25395766	91.60	19789227	77.92	4849174	19.09	2.98
		42ºC	6A	32771150	30503930	93.08	23811250	78.06	10438168	18.96	2.98
			6B	29703647	27717207	93.31	23328665	84.17	3671983	13.25	2.58
			6C	25487141	23755944	93.21	23755944	82.69	3474245	14.62	2.68
			Average	29320646	27325694	93.20	23631953	81.64	5861465	15.61	2.74
**CL153**	eCO_2	25ºC	7A	29807104	27910922	93.64	23588319	84.51	3616849	12.96	2.53
			7B	26162690	24625490	94.12	20827395	84.58	3168514	12.87	2.56
			7C	25883732	24238764	93.64	20078961	82.84	3461021	14.27	2.88
			Average	27284509	25591725	93.80	21498225	83.97	3415461	13.36	2.65
		37ºC	12A	33425021	30805515	92.16	25103564	81.49	4788867	15.54	2.97
			12B	29484379	27221963	92.33	22151760	81.37	4280142	15.72	2.90
			12C	28295180	26140159	92.38	21520950	82.33	3846870	14.71	2.96
			Average	30401527	28055879	92.29	22925424	81.73	4305293	15.32	2.94
		42ºC	8A	21937614	20616287	93.98	16335188	79.23	3666109	17.78	2.98
			8B	23329397	21918759	93.95	17303810	78.95	3946818	18.00	3.05
			8C	29033721	27027236	93.09	20238536	74.88	5928344	21.94	3.18
			Average	24766911	23187427	93.67	17959178	77.68	4513757	19.24	3.07
**Average CL153**				27647487	25688308	92.95	20818655	80.12	4620426	17.01	2.86
**Average**				**28346097**	**26309087**	**92.85**	**21696851**	**81.49**	**4205646**	**15.31**	**3.17**

Table S2. Number of expressed genes, number of DEGs found by DESeq2 and by edgeR analyses, number of overlapping DEGs from both analyses (overlapping: % between brackets). Plants were sub-mitted to control temperature at 25/20 °C (25 °C) and two different stress temperatures, 37/28 °C (37 °C) and 42/30 °C (42 °C), and grown in either 380 μL L−1 (aCO2) or 700 μL L−1 (eCO2). Differentially expressed genes (DEGs) correspond to the number of DEGs found in the two stress temperature treatments in comparison with the control temperature (respectively, 42 °C: 42/30 °C vs. 25/20 °C and 37 °C: 37/28 °C vs. 25/20 °C).

Genotype	Temperature	[CO2]	Expressed genes		DEGs		
			Treatment	Control	DESeq2	edgeR	Overlapping (%)
ICATU	42°C	aCO_2	21479	21714	12496	12729	11997 (95.12%)
	37°C	aCO_2	21324	21714	10726	11775	10485 (93.20%)
	42°C	eCO_2	20509	21659	13505	14132	13134 (95.05%)
	37°C	eCO_2	21319	21659	9669	11047	9545 (92.15%)
Average ICATU			21158	21687	11599	12421	11290 (94.00%)
CL153	42°C	aCO_2	20406	20728	10801	11921	10565 (92.99%)
	37°C	aCO_2	20500	20728	8351	9845	8240 (90.57%)
	42°C	eCO_2	19943	21186	12399	13254	12115 (94.45%)
	37°C	eCO_2	20700	21186	11414	9957	9795 (91.67%)
Average CL153			20387	20957	10741	11244	10179 (92.60%)
**Total average**			**20773**	**21322**	**11170**	**11833**	**10735 (93.34%)**

Table S3. Effect of temperature enhancement in Icatu and CL153 at ambient and elevated CO_2. Numbers indicate total differentially expressed genes (DEGs), GO annotated DEGs at 42 °C and at 37 °C, and the respective number of up- and down-regulated GO annotated DEGs at each temperature and CO_2 level (aCO_2: 380 μL L^{-1}; or eCO_2: 700 μL L^{-1}). DEGs correspond to the number of DEGs found in each stress temperature treatment in comparison with the control temperature (42 °C: 42/30 °C vs. 25/20 °C and 37 °C: 37/28 °C vs. 25/20 °C).

	[CO_2]	Temperature	Total DEGs			GO annotated DEGs		
			**All**	**Up**	**Down**	**All**	**Up**	**Down**
Icatu	aCO_2	42°C	11997	6027	5970	8786	4227	4559
		37°C	10485	5397	5088	7735	3819	3916
	eCO_2	42°C	13134	6355	6779	9676	4501	5175
		37°C	9545	4826	4719	7185	3527	3658
CL153	aCO_2	42°C	10565	5346	5219	7808	3828	3980
		37°C	8240	4269	3971	6151	3157	2994
	eCO_2	42°C	12115	5885	6230	8936	4140	4796
		37°C	9795	5002	4793	7303	3620	3683

Table S4. Significantly (FDR < 0.01) enriched gene ontology (GO) terms of the three main categories—biological process (GO:BP), molecular function (GO:MF), and cellular component (GO:CC)—among up-regulated, differentially expressed genes (DEGs), considering the effect of supra-optimal temperatures at 42/30 °C (42 °C) and 37/28 °C (37 °C) in Icatu and CL153 plants grown in either 380 μL L^{-1} aCO_2 or 700 μL L^{-1} eCO_2. DEGs were ranked by increasing log_2 fold change (FC) and an over-representation analysis (ORA) was performed using gProfiler, against *Coffea canephora* functional annotation. Counts indicate the total number of DEGs annotated with each GO term.

Treatment	Category	GO ID	GO Description	FDR	Counts
**Icatu**					
**37**°C-aCO_2	GO:MF	GO:0016168	chlorophyll binding	4.96E-06	12
	GO:MF	GO:0004869	cysteine-type endopeptidase inhibitor activity	1.07E-03	10
	GO:MF	GO:0010333	terpene synthase activity	7.45E-03	6
	GO:MF	GO:0008443	phosphofructokinase activity	8.42E-03	2
	GO:BP	GO:0015979	photosynthesis	6.24E-07	36
	GO:BP	GO:0019684	photosynthesis, light reaction	3.57E-05	18
	GO:BP	GO:0018298	protein-chromophore linkage	2.37E-03	12
	GO:CC	GO:0009521	photosystem	3.36E-05	28
	GO:CC	GO:0009579	thylakoid	2.79E-04	32
**37**°C-eCO_2	GO:MF	GO:0004866	endopeptidase inhibitor activity	4.50E-06	10
	GO:MF	GO:0016168	chlorophyll binding	1.02E-04	14
	GO:BP	GO:0015979	photosynthesis	1.88E-06	34
	GO:BP	GO:0010951	negative regulation of endopeptidase activity	1.96E-04	10
	GO:BP	GO:0006457	protein folding	3.43E-04	18
	GO:CC	GO:0009521	photosystem	5.84E-06	28
	GO:CC	GO:0009579	thylakoid	4.49E-04	28
42°C-aCO_2	GO:MF	GO:0031072	heat shock protein binding	7.88E-03	5
	GO:BP	GO:0006457	protein folding	4.34E-03	27
42°C-eCO_2	GO:MF	GO:0003723	RNA binding	4.45E-11	345
	GO:MF	GO:0051082	unfolded protein binding	1.11E-04	14
	GO:BP	GO:0006457	protein folding	2.18E-06	25

	GO:BP	GO:0042026	protein refolding	2.65E-04	8
**CL153**					
**37**°C-aCO_2	GO:BP	GO:0015979	photosynthesis	1.00E-05	39
	GO:CC	GO:0009521	photosystem	4.76E-04	22
	GO:CC	GO:0009579	thylakoid	1.13E-03	26
**37**°C-aCO_2	GO:MF	GO:0016168	chlorophyll binding	2.39E-06	12
	GO:MF	GO:0051082	unfolded protein binding	7.85E-04	8
	GO:BP	GO:0015979	photosynthesis	2.50E-11	33
	GO:BP	GO:0006457	protein folding	1.73E-05	16
	GO:BP	GO:0019684	photosynthesis, light reaction	3.25E-04	13
	GO:BP	GO:0018298	protein-chromophore linkage	1.24E-03	11
	GO:CC	GO:0009521	photosystem	9.50E-11	28
	GO:CC	GO:0009579	thylakoid	3.17E-10	32
	GO:CC	GO:0031984	organelle subcompartment	5.24E-03	12
42°C-aCO_2	GO:MF	GO:0051082	unfolded protein binding	2.01E-04	18
	GO:BP	GO:0006457	protein folding	2.52E-04	31
42°C-eCO_2	GO:MF	GO:0051082	unfolded protein binding	4.74E-07	12
	GO:MF	GO:0003723	RNA binding	1.76E-06	310
	GO:BP	GO:0006457	protein folding	4.15E-10	23
	GO:BP	GO:0042026	protein refolding	7.36E-05	8

Table S5. Significantly (FDR < 0.01) enriched gene ontology (GO) terms of the three main categories—biological process (GO:BP), molecular function (GO:MF), and cellular component (GO:CC)—among down-regulated, differentially expressed genes (DEGs), considering the effect of supra-optimal temperatures at 42/30 °C (42 °C) and 37/28 °C (37 °C) in Icatu and CL153 plants grown in either 380 μL L^{-1} aCO_2 or 700 μL L^{-1} eCO_2. DEGs were ranked by increasing log_2 fold change (FC) and an over-representation analysis (ORA) was performed using gProfiler, against *Coffea canephora* functional annotation. Counts indicate the total number of DEGs annotated with each GO term.

Treatment	Category	GO ID	GO Description	FDR	Counts
**Icatu**					
37°C-aCO_2	GO:MF	GO:0003968	RNA-directed 5'-3' RNA polymerase activity	3.30E-03	7
	GO:BP	GO:0006270	DNA replication initiation	3.52E-03	7
	GO:CC	GO:0042555	MCM complex	1.03E-03	6
37°C-eCO_2	GO:MF	GO:0003777	microtubule motor activity	1.63E-06	22
	GO:MF	GO:0008017	microtubule binding	2.34E-06	33
	GO:MF	GO:0004857	enzyme inhibitor activity	1.12E-04	13
	GO:MF	GO:0033293	monocarboxylic acid binding	3.64E-03	5
	GO:BP	GO:0007018	microtubule-based movement	1.39E-03	22
	GO:BP	GO:0006928	movement of cell or subcellular component	1.39E-03	22
	GO:BP	GO:0043086	negative regulation of catalytic activity	8.82E-03	13
42°C-aCO_2	GO:MF	GO:0008017	microtubule binding	2.18E-07	45
	GO:MF	GO:0005507	copper ion binding	9.18E-05	15
	GO:MF	GO:0003777	microtubule motor activity	1.65E-04	27
	GO:MF	GO:0016682	oxidoreductase activity, acting on diphenols and related substances as donors, oxygen as acceptor	4.86E-04	13
	GO:BP	GO:0009664	plant-type cell wall organization	5.86E-03	7
42°C-eCO_2	GO:BP	GO:0046274	lignin catabolic process	9.14E-05	8
	GO:BP	GO:0019748	secondary metabolic process	8.65E-04	6
**CL153**					
37°C-eCO_2	GO:MF	GO:0016682	oxidoreductase activity, acting on diphenols and related substances as donors, oxygen as acceptor	1.51E-06	12
	GO:MF	GO:0016838	carbon-oxygen lyase activity, acting on phosphates	1.43E-05	8
	GO:MF	GO:0016705	oxidoreductase activity, acting on paired donors, with incorporation or reduction of molecular oxygen	1.29E-04	10
	GO:MF	GO:0005506	iron ion binding	2.36E-04	10

	GO:MF	GO:0016762	xyloglucan:xyloglucosyl transferase activity	9.02E-04	11
	GO:MF	GO:0016491	oxidoreductase activity	9.93E-04	18
	GO:MF	GO:0071949	FAD binding	2.49E-03	12
	GO:MF	GO:0000287	magnesium ion binding	2.52E-03	9
	GO:MF	GO:0020037	heme binding	2.70E-03	9
	GO:MF	GO:0046906	tetrapyrrole binding	3.57E-03	9
	GO:MF	GO:0004497	monooxygenase activity	6.36E-03	7
42°C-aCO_2	GO:MF	GO:0005215	transporter activity	6.97E-06	61
	GO:MF	GO:0005337	nucleoside transmembrane transporter activity	5.10E-04	2
	GO:MF	GO:0042910	xenobiotic transmembrane transporter activity	1.34E-03	12
	GO:MF	GO:1901505	carbohydrate derivative transmembrane transporter activity	4.09E-03	2
	GO:BP	GO:0072530	purine-containing compound transmembrane transport	9.92E-04	2
42°C-eCO_2	GO:MF	GO:0016682	oxidoreductase activity, acting on diphenols and related substances as donors, oxygen as acceptor	3.66E-04	13
	GO:MF	GO:0008146	sulfotransferase activity	4.04E-03	11
	GO:MF	GO:0005509	calcium ion binding	5.93E-03	13
	GO:MF	GO:0016762	xyloglucan:xyloglucosyl transferase activity	8.20E-03	7

Table S6. Changes in Icatu and CL153 significant differential expression genes (DEGs) related to photosynthesis as a response to supra-optimal temperatures at 42/30 °C (42 °C) and 37/28 °C (37 °C), in plants grown in either 380 μL L^{-1} aCO_2 or 700 μL L^{-1} eCO_2 according to *Coffea canephora* genome functional annotation, downloaded from Coffee Genome Hub (http://coffee- genome.org/download, 13 July 2019). The searched gene ontology (GO) biological processes included: “photosynthesis”, “chlorophyll metabolic process”, “ribulose-bisphosphate carboxylase activity”, “antioxidant activity”, “lipid metabolic process (LOX, FAD)”, “cellular respiration”, “malate dehydrogenase activity”, and “pyruvate kinase activity”, as well as their direct child terms. Molecular functions were retrieved from UniprotKB (N/A: genes unannotated with molecular function GO terms). Blue indicates down-regulated while red up-regulated DEGs.

**Genotype**			**Icatu**				**CL153**			
**CO2**			**380**		**700**		**380**		**700**	
**Temperature**			**IT**	**HI**	**IT**	**HI**	**IT**	**HI**	**IT**	**HI**
**Gene ID**	**Protein Name**	**Molecular Function**								
**Photosynthesis**										
Cc07_g03250	ABC transporter B family member 19	ATP binding	0.04	-1.53	0.05	-0.83		-0.55	-0.13	-1.62
Cc01_g06000	Magnesium-chelatase subunit chlD, chloroplastic		-0.09	-0.91	-0.07	-0.66			0.05	
Cc07_g18500	Magnesium-chelatase subunit chlI, chloroplastic		-0.04	-1.09		-1.03		-0.61	0.09	-0.46
Cc01_g11930	Phototropin-1		0.1		0.05	-0.74	0.08		0.06	-0.91
Cc06_g00490	Probable serine/threonine-protein kinase WNK4			-2.84		-3.82	-0.22	-5.83	-0.45	-4.69
Cc02_g25110	Protein kinase G11A			0.33		0.24				
Cc07_g01630	Protein kinase PVPK-1			-0.64	-0.08	-1.19	0.06	0.38		
Cc02_g39010	Putative Protein SUPPRESSOR OF PHYA-105 1		-0.09			-1.04	-0.17			
Cc06_g00310	Serine/threonine-protein kinase STN8, chloroplastic		-0.02	-1.06	-0.11	-2.48		-0.89	0.04	-1.07
Cc10_g00010	Uncharacterized aarF domain-containing protein kinase At1g79600, chloroplastic		0.05	0.7	0.1	1.04	0.06	0.54	0.17	1.35
Cc08_g08480	Uncharacterized protein		-0.1		-0.2				0.31	2.2
Cc05_g09650	Chlorophyll a-b binding protein 1, chloroplastic	chlorophyll binding	0.21	-3.31	0.35	-3.36	0.2		0.43	1.38
Cc05_g12720	Chlorophyll a-b binding protein 13, chloroplastic		0.18	-0.55	0.08	-1.56	0.24	0.89	0.28	0.77
Cc07_g00260	Chlorophyll a-b binding protein 13, chloroplastic		0.16	-0.72	0.1	-1.56				
Cc09_g09010	Chlorophyll a-b binding protein 21, chloroplastic		0.14	-1.9	0.18	-1.95	0.12		0.25	0.6
Cc09_g09030	Chlorophyll a-b binding protein 21, chloroplastic		0.08	-1.58	0.09	-1.79	0.07		0.2	0.51

Genotype			Icatu				CL153			
**CO2**			380		700		380		700	
**Temperature**			IT	HI	IT	HI	IT	HI	IT	HI
Cc09_g09020	Chlorophyll a-b binding protein 21, chloroplastic		0.13	-1.2	0.12	-1.53	0.06		0.13	
Cc09_g09500	Chlorophyll a-b binding protein 36, chloroplastic		0.09	-0.89	0.16	-0.79	0.03	-0.36	0.22	0.8
Cc04_g16410	Chlorophyll a-b binding protein 4, chloroplastic		0.16		0.21	-0.23	0.2	1.19	0.39	1.98
Cc09_g02010	Chlorophyll a-b binding protein 6A, chloroplastic		0.14		0.23	0.16	0.2	1.29	0.42	2.25
Cc05_g09930	Chlorophyll a-b binding protein 8, chloroplastic		0.2	0.38	0.16	-0.53	0.28	1.48	0.33	1.57
Cc02_g21720	Chlorophyll a-b binding protein CP24 10A, chloroplastic		0.13	-0.54	0.15	-0.93	0.16	0.66	0.33	1.29
Cc10_g16210	Chlorophyll a-b binding protein CP26, chloroplastic		0.13	-0.4	0.18	-0.55	0.21	0.95	0.4	1.66
Cc06_g01460	Chlorophyll a-b binding protein CP29.2, chloroplastic		0.13		0.19	-0.2	0.13	0.43	0.31	1.39
Cc10_g04190	Chlorophyll a-b binding protein P4, chloroplastic		0.06	-1.02	0.05	-1.6		-0.56	0.15	
Cc06_g12480	Chlorophyll a-b binding protein, chloroplastic		-0.06	-1.79	-0.09	-2.2		-0.82	0.04	-1.16
Cc11_g16910	Chlorophyll a-b binding protein, chloroplastic		0.14		0.17	-0.14	0.22	1.2	0.31	1.44
Cc10_g00140	Putative Chlorophyll a-b binding protein 4, chloroplastic		-0.07	-0.88	-0.07	-1.12		-0.27	0.14	0.38
Cc02_g00040	Putative RNA polymerase sigma-B factor	DNA binding	0.13	0.65	0.08	0.21	0.24	1.68	0.24	1.59
Cc00_g04770	Cryptochrome DASH, chloroplastic/mitochondrial	DNA photolyase activity			-0.09	-1	-0.12	-1.04		-1.26
Cc05_g13170	PsbQ-like 1	electron transfer activity	-0.09	-1.87	-0.11	-3.03		-0.46	0.08	-1.04
Cc08_g15460	CAAX amino terminal protease family protein	hydrolase activity	0.11	1.34	0.05	0.9		0.29		
Cc10_g14740	Fructose-1,6-bisphosphatase, cytosolic		0.11		0.05	-0.86	0.23	0.69	0.37	1.2
Cc05_g09330	haloacid dehalogenase (HAD) superfamily protein		-0.08					0.56		
Cc02_g18440	Pentatricopeptide repeat-containing protein At2g15820		0.04		0.05				0.15	0.49
Cc06_g07060	Probable glutamate carboxypeptidase 2		-0.1	-1.31	0.12		-0.2	-1.7		
Cc07_g02170	Protease Do-like 1, chloroplastic		0.12	0.6	0.11	0.22	0.08	0.5	0.12	0.4
Cc01_g20240	Protease Do-like 2, chloroplastic		-0.1	-0.63	-0.04		-0.05	-0.37		

Genotype			Icatu				CL153			
**CO2**			380		700		380		700	
**Temperature**			IT	HI	IT	HI	IT	HI	IT	HI
Cc02_g01340	Protein phosphatase 2C 57			-0.93	-0.19	-2.53	0.09	-0.28	-0.03	-1.09
Cc03_g00090	APO protein 1, chloroplastic	ion binding	0.04	0.32	0.1	0.34	0.15	1.08	0.31	1.52
Cc07_g04520	Auxin transport protein BIG		0.04	0.89	0.05	1.43		1	0.1	1.95
Cc05_g00840	Oxygen-evolving enhancer protein 2, chloroplastic		0.12		0.15	-0.28	0.18	0.87	0.31	1.53
Cc02_g11770	Oxygen-evolving enhancer protein 3-2, chloroplastic		0.16		0.21		0.19	0.69	0.3	1.29
Cc08_g01270	Peroxisome biogenesis factor 10							0.4	0.15	0.82
Cc06_g20260	Peroxisome biogenesis protein 2			0.67		0.56	0.07	0.71	0.07	0.88
Cc11_g16230	Photosystem II core complex proteins psbY, chloroplastic		0.13	-0.38	0.14	-0.87	0.2	0.69	0.27	0.96
Cc02_g31060	Photosystem II reaction center PsbP family protein		0.11	0.58	0.17	0.54	0.25	1.49	0.22	0.98
Cc02_g33910	Predicted protein (Fragment)		-0.04	-0.84		-0.77		-0.67		
Cc02_g35130	PsbP domain-containing protein 1, chloroplastic		0.05	-0.75		-1.32	0.08		0.06	-0.33
Cc10_g00260	PsbP domain-containing protein 3, chloroplastic			-0.97	-0.03	-1.69		-1.19	0.08	-1.03
Cc10_g12990	PsbP domain-containing protein 4, chloroplastic		0.16	0.63	0.12		0.21	1.28	0.23	1.25
Cc08_g08290	PsbP domain-containing protein 5, chloroplastic			-0.56	0.07				0.23	1.25
Cc06_g20190	PsbP domain-containing protein 6, chloroplastic		0.03	-1.1	0.06	-1.39	0.08	-0.53	0.22	
Cc11_g04720	PsbP-like protein 1, chloroplastic		-0.02	-1.16	-0.05	-1.72	0.06	-0.31	0.15	
Cc11_g12760	PsbQ-like 2		-0.06	-1.75	-0.11	-2.32	0.09	-0.55	0.08	-1
Cc07_g09880	Putative Oxygen-evolving enhancer protein 3-2, chloroplastic					-1.58				
Cc02_g08380	Putative PsbP-like protein 1, chloroplastic		-0.05	-1.55	-0.06	-1.68		-0.81	0.08	-0.45
Cc02_g27800	Thylakoid lumenal 19 kDa protein, chloroplastic		0.15	0.95	0.19	1	0.21	1.07	0.22	1.04
Cc04_g03080	Putative Glyoxylate reductase	oxidoreductase activity	0.06			-0.39	0.11	0.65	0.07	0.2
Cc00_g24160	Putative dihydrodipicolinate reductase 3, chloroplastic		-0.07	-0.82	-0.1	-1.12	-0.2	-1.85	-0.16	-1.95
Cc02_g30160	Geranylgeranyl diphosphate reductase, chloroplastic		-0.2		-0.21					

**Genotype**			**Icatu**				**CL153**			
**CO2**			**380**		**700**		**380**		**700**	
**Temperature**			**IT**	**HI**	**IT**	**HI**	**IT**	**HI**	**IT**	**HI**
Cc04_g16560	Geranylgeranyl diphosphate reductase, chloroplastic		0.14		0.12	-0.61	0.16	0.78	0.17	0.69
Cc06_g22740	Magnesium-protoporphyrin IX monomethyl ester [oxidative] cyclase, chloroplastic		0.19		0.14	-1.06	0.26	1.01	0.26	0.87
Cc07_g05350	Oxygen-evolving enhancer protein 1, chloroplastic		0.14		0.17		0.14	0.63	0.28	1.48
Cc05_g12370	Protochlorophyllide reductase, chloroplastic		0.06	-1.46	0.23		-0.13	-1.42		-0.28
Cc05_g06850	Protochlorophyllide reductase, chloroplastic		0.16		0.22	0.35	0.12	0.59	0.3	1.46
Cc00_g15710	Ribulose bisphosphate carboxylase small chain SSU11A, chloroplastic		-0.03	-1.3		-1.5	0.07	-0.47	0.12	-0.54
Cc10_g07160	Cryptochrome-1	photoreceptor activity	0.11	0.93	0.13	1.05	0.1	0.79	0.16	1.19
Cc03_g05480	Cryptochrome-2		-0.07	-0.85	-0.03	-1.07	-0.09	-1.03	-0.14	-1.26
Cc02_g36930	Phytochrome A1				0.05	0.49		0.44	0.15	1.2
Cc07_g19320	Putative Probable E3 ubiquitin-protein ligase HERC4		-0.1	-0.5	-0.15	-0.5	-0.08		-0.09	-0.41
Cc04_g00370	proton gradient regulation 7	protein-membrane adaptor activity	-0.04	-0.74		-0.6	0.05	0.47	0.12	0.56
Cc05_g16390	Nuclear cap-binding protein subunit 1	RNA binding		0.5	0.04	0.78		0.34	0.08	0.73
Cc06_g10290	SNW domain-containing protein 1		-0.04	0.4		1.2	-0.11	-0.36		0.51
Cc05_g14900	Nuclear transcription factor Y subunit B	transcription coregulator activity	0.13	1.71	0.11	1.77		1.02	0.15	1.51
Cc04_g01030	Unknown protein		-0.03			0.74	-0.07		0.07	1.14
Cc06_g16120	methyltransferases	transferase activity					-0.1	-0.48		
Cc01_g10030	Magnesium transporter MRS2-A, chloroplastic	transporter activity		-0.53	-0.03	-0.99	0.08		0.12	
Cc01_g21780	Protein translocase subunit SecA, chloroplastic					-0.27		-0.41	0.11	0.36
Cc06_g16240	C2 calcium/lipid-binding plant phosphoribosyltransferase family protein	N/A	-0.15	-3.14	-0.12	-3.87	-0.24	-2.51	-0.16	-2.43
Cc11_g15850	chloroplast sensor kinase		0.03	0.69	-0.06	0.26	0.11	0.56	0.05	0.61

**Genotype**		**Icatu**				**CL153**			
**CO2**		**380**		**700**		**380**		**700**	
**Temperature**		**IT**	**HI**	**IT**	**HI**	**IT**	**HI**	**IT**	**HI**
Cc04_g15980	Epstein-Barr nuclear antigen, putative	0.06	1.25	0.05	1	0.08	0.68	0.08	0.74
Cc02_g28180	Ferredoxin-thioredoxin reductase, variable chain	0.04			-1.08	0.13		0.07	-0.5
Cc10_g15180	one-helix protein 2	0.08			-0.99	0.16	0.34	0.11	
Cc03_g03590	Photosystem I reaction center subunit II, chloroplastic	0.14	-0.47	0.17	-0.73	0.21	0.76	0.37	1.6
Cc09_g06610	Photosystem I reaction center subunit III, chloroplastic	0.09	-0.73	0.13	-0.79	0.09	0.32	0.2	0.69
Cc01_g17500	Photosystem I reaction center subunit IV A, chloroplastic	0.12		0.13	-0.55	0.13	0.43	0.23	0.97
Cc10_g12590	Photosystem I reaction center subunit N, chloroplastic	0.16		0.13	-0.41	0.22	0.99	0.19	0.79
Cc09_g08490	Photosystem I reaction center subunit psaK, chloroplastic	0.12	-0.64	0.05	-1.82	0.2	0.48	0.16	
Cc04_g03050	Photosystem I reaction center subunit VI, chloroplastic	0.15	-0.68	0.17	-1.11	0.21	1	0.35	1.47
Cc01_g15890	Photosystem I reaction center subunit XI, chloroplastic	0.1	-0.76	0.15	-0.78	0.18	0.82	0.31	1.23
Cc01_g08780	photosystem I subunit O	0.15	-0.73	0.23	-0.66	0.17	0.71	0.34	1.59
Cc03_g03760	photosystem II 11 kDa protein-related	-0.07	-1.27		-1.19	-0.15	-1.67		-1.07
Cc01_g17770	Photosystem II reaction center PSB28 protein, chloroplastic	0.1			-1.04	0.13		0.16	
Cc01_g18800	Photosystem II reaction center W protein, chloroplastic	0.14		0.14	-0.64	0.22	1.05	0.3	1.41
Cc06_g11950	photosystem II subunit X	0.22		0.24	-0.83	0.28	0.99	0.34	1.47
Cc08_g01300	photosystem II subunit X	0.11	-0.5	0.05	-1.64	0.2	0.42	0.22	0.36
Cc01_g20770	Phytochrome A-associated F-box protein	0.07	0.82				0.51	-0.1	-0.6
Cc10_g15270	Protein GIGANTEA	-0.04		0.04	1.46	-0.46	-2.05	-0.33	-0.56
Cc08_g05240	Protein PAM68, chloroplastic		-0.97	0.07	-1.5		-0.69	-0.07	-1.27
Cc05_g06070	Protein THYLAKOID FORMATION1, chloroplastic	0.13		0.15		0.18	1.06	0.31	1.51
Cc11_g02780	Protein XAP5 CIRCADIAN TIMEKEEPER	0.03	0.74		0.53	0.04	0.64	0.06	0.82
Cc04_g02320	Putative Predicted protein	-0.22	-3.1	-0.21	-2.94	0.15	1.22	0.16	
Cc05_g11580	Putative Predicted protein	-0.19	-2.9	0.07	-0.69	-0.31	-2.08	-0.09	-0.93

**Genotype**			**Icatu**				**CL153**			
**CO2**			**380**		**700**		**380**		**700**	
**Temperature**			**IT**	**HI**	**IT**	**HI**	**IT**	**HI**	**IT**	**HI**
Cc02_g16780	Putative Protein AF-9 homolog		-0.06				-0.22	-1.67	-0.12	-0.9
Cc07_g16050	Putative Protein CPR-5								0.06	0.68
Cc08_g10110	Putative Protein EARLY FLOWERING 3		0.09	1.89	0.22	3.2	0.05	1.76	0.32	3.28
Cc11_g13080	Putative Protein PHYTOCHROME KINASE SUBSTRATE 3		0.1	-1.22		-2.81	0.21	1.08		-0.69
Cc06_g02340	Putative uncharacterized protein		0.08			-1.48	0.25	0.84	0.21	
Cc02_g17350	Putative uncharacterized protein		0.06	0.63	-0.07	-0.28	0.2	1.11		
Cc09_g04620	Putative uncharacterized protein		0.07		0.09	-0.29	0.08	0.2	0.19	0.66
Cc04_g02050	Putative uncharacterized protein					-0.62			0.07	
Cc06_g22680	Putative Uncharacterized protein C9orf78		0.11	0.96	0.07	0.71				
Cc09_g04630	Putative unknown protein		0.18	3.5		3.13				
Cc01_g15410	Root phototropism protein 2		0.41	2.81	0.14	1.07	0.45	2.3	0.05	-0.26
Cc06_g08290	Thylakoid lumenal 16.5 kDa protein, chloroplastic		-0.06	-0.81		-0.39	-0.06	-0.76		-0.82
Cc05_g06750	Thylakoid lumenal protein At1g03610, chloroplastic		0.13		0.12	-0.42	0.26	0.9	0.27	0.7
Cc02_g09420	Uncharacterized protein		0.1	-0.59	0.23	-0.49		-1.56	0.07	-1.19
Cc07_g02300	Uncharacterized protein		0.03		0.05		0.09	0.4	0.14	0.36
Cc08_g16100	Unknown protein		-0.05	-1.18	-0.04	-1.39			0.1	
Cc02_g32670	UPF0603 protein At1g54780, chloroplastic			-1.85		-2.5	0.08	-0.67	0.12	-0.51
Cc00_g26590	YEATS family protein		-0.16	-0.97	-0.18	-1.66	-0.19	-1.29	-0.16	-1.37
**Chlorophyll metabolic process**										
Cc01_g06000	Magnesium-chelatase subunit chlD, chloroplastic	ATP binding	-0.09	-0.91	-0.07	-0.66			0.05	
Cc07_g18500	Magnesium-chelatase subunit chlI, chloroplastic		-0.04	-1.09		-1.03		-0.61	0.09	-0.46
Cc06_g17100	Putative Magnesium-chelatase subunit H		0.15	-0.35	0.05	-1.39	0.23	0.8	0.15	0.36
Cc05_g09650	Chlorophyll a-b binding protein 1, chloroplastic	chlorophyll binding	0.21	-3.31	0.35	-3.36	0.2		0.43	1.38
Cc05_g12720	Chlorophyll a-b binding protein 13, chloroplastic		0.18	-0.55	0.08	-1.56	0.24	0.89	0.28	0.77

Genotype			Icatu				CL153			
**CO2**			**380**		**700**		**380**		**700**	
**Temperature**			**IT**	**HI**	**IT**	**HI**	**IT**	**HI**	**IT**	**HI**
Cc07_g00260	Chlorophyll a-b binding protein 13, chloroplastic		0.16	-0.72	0.1	-1.56				
Cc09_g09010	Chlorophyll a-b binding protein 21, chloroplastic		0.14	-1.9	0.18	-1.95	0.12		0.25	0.6
Cc09_g09030	Chlorophyll a-b binding protein 21, chloroplastic		0.08	-1.58	0.09	-1.79	0.07		0.2	0.51
Cc09_g09020	Chlorophyll a-b binding protein 21, chloroplastic		0.13	-1.2	0.12	-1.53	0.06		0.13	
Cc09_g09500	Chlorophyll a-b binding protein 36, chloroplastic		0.09	-0.89	0.16	-0.79	0.03	-0.36	0.22	0.8
Cc04_g16410	Chlorophyll a-b binding protein 4, chloroplastic		0.16		0.21	-0.23	0.2	1.19	0.39	1.98
Cc09_g02010	Chlorophyll a-b binding protein 6A, chloroplastic		0.14		0.23	0.16	0.2	1.29	0.42	2.25
Cc05_g09930	Chlorophyll a-b binding protein 8, chloroplastic		0.2	0.38	0.16	-0.53	0.28	1.48	0.33	1.57
Cc02_g21720	Chlorophyll a-b binding protein CP24 10A, chloroplastic		0.13	-0.54	0.15	-0.93	0.16	0.66	0.33	1.29
Cc10_g16210	Chlorophyll a-b binding protein CP26, chloroplastic		0.13	-0.4	0.18	-0.55	0.21	0.95	0.4	1.66
Cc06_g01460	Chlorophyll a-b binding protein CP29.2, chloroplastic		0.13		0.19	-0.2	0.13	0.43	0.31	1.39
Cc10_g04190	Chlorophyll a-b binding protein P4, chloroplastic		0.06	-1.02	0.05	-1.6		-0.56	0.15	
Cc06_g12480	Chlorophyll a-b binding protein, chloroplastic		-0.06	-1.79	-0.09	-2.2		-0.82	0.04	-1.16
Cc11_g16910	Chlorophyll a-b binding protein, chloroplastic		0.14		0.17	-0.14	0.22	1.2	0.31	1.44
Cc10_g00140	Putative Chlorophyll a-b binding protein 4, chloroplastic		-0.07	-0.88	-0.07	-1.12		-0.27	0.14	0.38
Cc06_g01120	Chlorophyll synthase, chloroplastic	chlorophyll synthetase activity	-0.08	-1.51	-0.05	-1.46			0.04	0.21
Cc03_g01230	Two-component response regulator ARR2	DNA binding	0.1	0.58			0.22	0.7	-0.07	-0.93
Cc05_g13170	PsbQ-like 1	electron transfer activity	-0.09	-1.87	-0.11	-3.03		-0.46	0.08	-1.04
Cc03_g04230	Chlorophyllase-2, chloroplastic	hydrolase activity	-0.17	-0.33	-0.04	0.64	-0.3	-1.53	-0.24	-1.4
Cc02_g01340	Protein phosphatase 2C 57			-0.93	-0.19	-2.53	0.09	-0.28	-0.03	-1.09
Cc01_g19080	Putative Chlorophyllase-1		0.06	-1.71	0.22	-2.49	0.12	0.19	0.1	

Genotype			Icatu				CL153			
**CO2**			**380**		**700**		**380**		**700**	
**Temperature**			**IT**	**HI**	**IT**	**HI**	**IT**	**HI**	**IT**	**HI**
Cc05_g09190	Serine/threonine-protein phosphatase 5			0.76		0.84	0.06	0.82	0.05	0.5
Cc02_g32140	Chlorophyll(ide) b reductase NOL, chloroplastic	oxidoreductase activity		-0.47		-0.38			0.1	0.45
Cc10_g11980	Chlorophyllide a oxygenase, chloroplastic		0.29	1.83	-0.04	-0.38	0.28	1.4	0.07	0.43
Cc02_g30160	Geranylgeranyl diphosphate reductase, chloroplastic		-0.2		-0.21					
Cc04_g16560	Geranylgeranyl diphosphate reductase, chloroplastic		0.14		0.12	-0.61	0.16	0.78	0.17	0.69
Cc06_g22740	Magnesium-protoporphyrin IX monomethyl ester [oxidative] cyclase, chloroplastic		0.19		0.14	-1.06	0.26	1.01	0.26	0.87
Cc06_g17670	NADPH-dependent thioredoxin reductase 3		-0.03	-0.73	-0.04	-0.94		-0.22		
Cc01_g10220	Pheophorbide a oxygenase, chloroplastic		0.21	2.56	0.26	2.53	0.21	1.82	0.36	2.34
Cc06_g09730	Probable chlorophyll(ide) b reductase NYC1, chloroplastic		0.05		0.04	0.52	-0.11	-0.5	-0.09	-0.22
Cc08_g06700	Protein TIC 55, chloroplastic		0.18	1.98	0.16	1.65	0.35	2.43	0.38	2.3
Cc05_g12370	Protochlorophyllide reductase, chloroplastic		0.06	-1.46	0.23		-0.13	-1.42		-0.28
Cc05_g06850	Protochlorophyllide reductase, chloroplastic		0.16		0.22	0.35	0.12	0.59	0.3	1.46
Cc01_g00670	Protochlorophyllide-dependent translocon component 52, chloroplastic		0.05		0.09	0.14	0.06	0.33	0.17	0.66
Cc03_g11370	Red chlorophyll catabolite reductase, chloroplastic		-0.04	-1.02		-1.14			0.16	0.65
Cc07_g00790	Sulfite oxidase		0.11		-0.05	-1.08	0.06			-0.49
Cc08_g15230	2-C-methyl-D-erythritol 2,4-cyclodiphosphate synthase, chloroplastic	phosphorus-oxygen lyase activity			0.06		0.04	0.53	0.12	0.7
Cc04_g00370	proton gradient regulation 7	protein-membrane adaptor activity	-0.04	-0.74		-0.6	0.05	0.47	0.12	0.56
Cc06_g21470	Putative Polyribonucleotide nucleotidyltransferase	RNA binding		0.32	-0.03	0.29	-0.07	-0.37		0.3
Cc01_g08780	photosystem I subunit O	N/A	0.15	-0.73	0.23	-0.66	0.17	0.71	0.34	1.59

**Genotype**			**Icatu**				**CL153**			
**CO2**			**380**		**700**		**380**		**700**	
**Temperature**			**IT**	**HI**	**IT**	**HI**	**IT**	**HI**	**IT**	**HI**
Cc10_g02580	Predicted protein		0.16	1.2	0.17	1.25	0.08	0.46	0.27	1.07
Cc05_g12410	Putative COP1-interacting protein 7			-1.89	-0.06	-1.85		-0.5	-0.16	-1.17
Cc01_g15580	Putative uncharacterized protein		0.04		0.11	0.79	0.05	0.49	0.14	0.91
Cc08_g16100	Unknown protein		-0.05	-1.18	-0.04	-1.39			0.1	
**RuBisCO**										
Cc04_g05490	Putative Ribulose bisphosphate carboxylase/oxygenase activase, chloroplastic	ATP binding	0.06	-0.74	0.06	-0.71	0.15	0.43	0.21	0.78
Cc04_g14500	Ribulose bisphosphate carboxylase/oxygenase activase 1, chloroplastic		0.68	5.22	0.59	3.3	0.98	6.51	1.26	6.8
Cc01_g10720	RuBisCO large subunit-binding protein subunit alpha, chloroplastic		0.23	1.88	0.26	2.58	0.22	1.5	0.47	3.21
Cc01_g00200	RuBisCO large subunit-binding protein subunit beta, chloroplastic		0.22	2.38	0.24	2.93	0.13	1.97	0.4	3.35
Cc04_g02750	RuBisCO large subunit-binding protein subunit beta, chloroplastic		0.05	1.2		1.85	0.07	0.66	0.11	1.78
Cc00_g15710	Ribulose bisphosphate carboxylase small chain SSU11A, chloroplastic	ribulose-bisphosphate carboxylase activity	-0.03	-1.3		-1.5	0.07	-0.47	0.12	-0.54
Cc02_g07500	Ribulose bisphosphate carboxylase/oxygenase activase 1, chloroplastic		0.11	-0.31		-1.48	0.18		0.13	-0.16
Cc11_g12670	Ribulose-1,5 bisphosphate carboxylase/oxygenase large subunit N-methyltransferase, chloroplastic	transferase activity	-0.05			-0.5			0.16	
Cc06_g08500	DnaJ/Hsp40 cysteine-rich domain superfamily protein	N/A	0.09			-0.5	0.17	0.4	0.16	0.18
**Antioxidant activity**										
Cc06_g12530	20 kDa chaperonin, chloroplastic	ATP binding	0.28	2.58	0.3	3.22	0.27	2.12	0.48	3.33
Cc09_g06000	sulfiredoxin		0.12	1.09	0.14	0.79	0.15	1.16	0.36	1.8
Cc04_g06610	alpha dioxygenase		-0.21	-2.19	0.09	-1.11	0.07		0.22	0.43

Genotype			Icatu				CL153			
CO_2			380		700		380		700	
Temperature			IT	HI	IT	HI	IT	HI	IT	HI
Cc10_g00570	Catalase	oxidoreductase activity	0.07	0.47	0.15	0.95	0.09	0.3	0.24	1.94
Cc07_g11710	Catalase isozyme 1		0.11		-0.08	-1.91	0.24	0.82	0.25	0.92
Cc07_g11720	Catalase isozyme 2		0.16		-0.08	-2.34	0.2	0.72		-0.3
Cc10_g00580	Catalase isozyme 3			-0.68	0.1	-0.28	-0.06	-0.96		-0.26
Cc06_g00570	Cationic peroxidase 1		-0.28	-3.16	-0.34	-3.58	-0.47	-2.91	-0.69	-4.92
Cc03_g07600	Glutathione reductase, chloroplastic (Fragment)		-0.03		-0.03				-0.05	-0.46
Cc01_g18820	Glutathione reductase, chloroplastic (Fragment)		-0.08	-1.12	-0.1	-1.67	-0.09	-1.12	-0.07	-1.32
Cc02_g08510	Glutathione S-transferase DHAR2		0.13	1.24	0.15	1.14	0.27	1.65	0.16	1.04
Cc06_g03490	L-ascorbate peroxidase 2, cytosolic			2.53		4.12	0.7	7.08	0.8	6.24
Cc01_g11800	L-ascorbate peroxidase 2, cytosolic				0.1	0.92	0.18	1	0.42	2.15
Cc02_g16260	L-ascorbate peroxidase 3, peroxisomal		-0.05		-0.08	-0.33		-0.61	0.03	-0.4
Cc07_g11020	L-ascorbate peroxidase 3, peroxisomal		-0.15	-2.63	-0.1	-3.64				
Cc10_g12080	L-ascorbate peroxidase T, chloroplastic				0.03					0.22
Cc06_g17670	NADPH-dependent thioredoxin reductase 3		-0.03	-0.73	-0.04	-0.94		-0.22		
Cc02_g18480	Peroxidase 10		-0.43	-3.24			-0.54	-5.62	-0.76	-4.52
Cc07_g05780	Peroxidase 10		-0.62	-3.04			-0.43	-6.56		
Cc11_g11190	Peroxidase 11								-0.25	-5.1
Cc02_g30380	Peroxidase 12		-0.13	-1.77	-0.11	-2.12			0.13	
Cc10_g15150	Peroxidase 12						-0.18	-1.37	-0.36	-2.04
Cc10_g15130	Peroxidase 12							-2.78	-0.46	-4.18
Cc10_g15140	Peroxidase 12								-0.28	-3.47
Cc06_g13090	Peroxidase 16		-0.28	-2.48	-0.23	-2.05	-0.14	-1.64	-0.12	-1.26
Cc07_g14740	Peroxidase 17		-0.32	-0.95			-0.74	-4	-0.72	-4.42
Cc08_g02130	Peroxidase 20		0.38	3.47	0.3	3.85	0.26			
Cc06_g08460	Peroxidase 25		0.32	1.61		-0.74				

Genotype		Icatu				CL153			
CO_2		380		700		380		700	
**Temperature**		**IT**	**HI**	**IT**	**HI**	**IT**	**HI**	**IT**	**HI**
Cc05_g04990	Peroxidase 3	-0.13	-3.65	-0.15	-3.42			-0.36	-3.52
Cc00_g17550	Peroxidase 3	-0.11	-2.18		-2.93	0.25	1.16	-0.11	-0.86
Cc05_g08480	Peroxidase 4	-0.04	-2.18		-0.98	-0.07	-1.06	-0.22	-1.93
Cc01_g15110	Peroxidase 4	-0.17	-3.52	-0.15	-2.09	-0.13	-1.3	-1.05	-2.89
Cc02_g05640	Peroxidase 4	0.1	-2.69	0.17	-4.07		-2.36	-0.26	-3.2
Cc09_g01700	Peroxidase 42	-0.03	-2.86	-0.1	-3.35	0.08		-0.08	-1.24
Cc02_g21960	Peroxidase 43		-2.9	-0.15	-3.76	0.14			-2.25
Cc00_g00940	Peroxidase 47		-2.4		-2.5				-1.45
Cc07_g17340	Peroxidase 5		-2.96						-4.68
Cc07_g02590	Peroxidase 63	-0.15	-3.1	-0.27	-3.22		-1.15	-0.17	-2.97
Cc07_g11210	Peroxidase 64		-6.02	-0.09	-4.92			-0.18	-1.95
Cc01_g08210	Peroxidase 64		0.76		1.59	-0.47	-3.32	-0.1	-1.65
Cc02_g03550	Peroxidase 66	-0.28	-4.69	-0.3	-5.76			-0.41	-3.62
Cc07_g10080	Peroxidase 72	-0.43	-3.23					-0.41	-3.08
Cc07_g06870	Peroxidase 73		-2.28	0.08	-1.13	0.24	-0.21	0.09	-0.95
Cc07_g02500	Peroxidase superfamily protein		0.7	-0.22	-0.89	-0.05	-1.34	-0.13	-1.65
Cc06_g12610	Peroxiredoxin Q, chloroplastic		-1.06		-1.27	0.11	0.25	0.17	0.46
Cc06_g09990	Peroxiredoxin-2B	0.06	0.43	0.09			0.15	0.11	
Cc06_g05140	Peroxiredoxin-2E, chloroplastic		-0.98		-1.03		-0.27	0.15	
Cc03_g03290	Peroxiredoxin-2F, mitochondrial						0.27		
Cc08_g07250	Phospholipid hydroperoxide glutathione peroxidase, chloroplastic		-0.65	0.04	-1.22	0.09	0.36	0.17	0.52
Cc00_g03790	Probable glutathione peroxidase 2				-0.2		-0.53	0.06	
Cc01_g19740	Probable glutathione peroxidase 4	0.05	0.77					-0.08	-0.28
Cc08_g14610	Probable glutathione peroxidase 8	-0.04	-0.5		-0.46			0.14	1.11

Genotype			Icatu				CL153			
CO2			380		700		380		700	
Temperature			IT	HI	IT	HI	IT	HI	IT	HI
Cc08_g14620	Probable glutathione peroxidase 8		0.03		0.05	0.58	0.14	0.74	0.18	1.28
Cc08_g14600	Probable phospholipid hydroperoxide glutathione peroxidase		0.07	0.59	0.03	-0.32	0.03	-0.68		-0.69
Cc06_g06390	Putative 1-cysteine peroxiredoxin 1		0.07		0.09	0.13	0.11	0.92	0.2	1.25
Cc08_g00640	Putative L-ascorbate peroxidase 2, cytosolic		0.06		0.05	-0.33		-0.3	0.1	
Cc01_g04080	Putative Long-chain-fatty-acid--AMP ligase FadD28							-1.76		
Cc08_g15360	Respiratory burst oxidase homolog protein A					-0.85	0.14	0.72	-0.23	-1.69
Cc04_g05580	Respiratory burst oxidase homolog protein C			-0.61	0.08		-0.11	-0.62		-0.62
Cc06_g12630	Respiratory burst oxidase homolog protein E		-0.27	-4.75	-0.08	-4.12	-0.29	-2.77	-0.21	-3.09
Cc03_g02460	Superoxide dismutase [Cu-Zn]		-0.04	-1.35	0.04	-1.32				-0.3
Cc02_g32280	Superoxide dismutase [Cu-Zn] 2		0.3	2.68	0.28	2.61	0.35	2.72	0.47	3.19
Cc06_g23170	Superoxide dismutase [Cu-Zn] 2		0.09	0.96	0.06	0.8	0.19	1.6	0.23	2.04
Cc06_g23140	Superoxide dismutase [Cu-Zn] 2			1.48	0.26	2.25				1.75
Cc01_g10280	Superoxide dismutase [Cu-Zn], chloroplastic			-0.6	-0.23	-2.28			-0.21	-0.9
Cc02_g04930	Superoxide dismutase [Fe], chloroplastic		0.04		0.07	0.37	0.11	0.86	0.19	1.16
Cc10_g09500	Superoxide dismutase [Fe], chloroplastic (Fragment)		-0.11	-1.7	-0.08	-1.94		-0.6	0.06	-0.68
Cc02_g05460	Superoxide dismutase [Mn], mitochondrial							-0.19	0.06	
Cc07_g12230	Thioredoxin reductase 2		-0.05	-0.35	-0.06	-0.47	-0.07	-0.43	-0.09	-0.45
Cc10_g14220	Thioredoxin superfamily protein		0.03		0.04	-0.4			0.14	
Cc10_g06720	Thylakoid lumenal 29 kDa protein, chloroplastic		0.06	-0.62	0.09	-0.59	0.06	0.29	0.27	1.33
Cc06_g18010	Unknown protein		0.08		0.1				1.39	
Cc06_g09870	Aspartate aminotransferase, cytoplasmic	transferase activity	-0.1					-1.87		
**Lipid metabolism: FAD/LOX**										
Cc01_g11420	Isovaleryl-CoA dehydrogenase 1, mitochondrial	ATP binding	0.14	1.95	0.2	2.29	-0.07	-0.19	0.37	2.53

Genotype			Icatu				CL153			
**CO2**			**380**		**700**		**380**		**700**	
**Temperature**			**IT**	**HI**	**IT**	**HI**	**IT**	**HI**	**IT**	**HI**
Cc01_g00140	Lysine--tRNA ligase		-0.07	-0.85		-0.38	-0.17	-1.18	-0.07	-0.64
Cc07_g21030	Lysine--tRNA ligase		-0.05		-0.04	0.21	-0.08	-0.56	-0.11	-0.67
Cc00_g09970	Nucleotidylyl transferase superfamily protein		-0.07	-0.42	-0.07	-0.75				
Cc00_g06160	Aldehyde oxidase 2	electron transfer activity		-3.68			-0.2	-2.18	-0.45	-2.51
Cc00_g06180	Aldehyde oxidase 4		-0.16		-0.09	-1.7	-0.31	-2.04	-0.19	-1.62
Cc01_g08420	Aldehyde oxidase 4		-0.06			-0.65	-0.12	-0.68		-0.43
Cc05_g13380	Electron transfer flavoprotein subunit alpha, mitochondrial		0.15	1.56	0.13	1.45	0.16	1.11	0.28	1.63
Cc07_g19610	Xanthine dehydrogenase 1		-0.17	-1.03	-0.12	-0.91			0.21	1.52
Cc07_g15410	Acyl-coenzyme A oxidase 2, peroxisomal	FAD binding	0.06	1.36	0.06	1.85			0.05	0.26
Cc05_g07930	Acyl-coenzyme A oxidase 3, peroxisomal				0.06	0.27				0.73
Cc00_g06140	Aldehyde oxidase 2								-0.53	-3.21
Cc10_g02380	Cytokinin dehydrogenase 5		-0.08	-4.95	-0.14	-5.32		-3.76	0.12	-3.4
Cc08_g01180	Cytokinin dehydrogenase 7		-0.04	-2.62	-0.12	-4.35	0.07	-1.66	0.15	-1.88
Cc02_g30100	Cytokinin dehydrogenase 9			-3.79		-3.62	0.16	-3.43		-3.63
Cc11_g06650	D-arabinono-1,4-lactone oxidase family protein				-0.46				-0.65	-4.63
Cc11_g06640	D-arabinono-1,4-lactone oxidase family protein								-0.52	-3.76
Cc06_g07030	D-lactate dehydrogenase [cytochrome], mitochondrial		0.06	0.6	0.1	0.59	0.04		0.2	0.44
Cc09_g06490	Delta(24)-sterol reductase			-1.04		-1.18		-0.78		-0.94
Cc06_g18590	L-galactono-1,4-lactone dehydrogenase, mitochondrial		-0.07	-0.51			-0.12	-0.32		0.68
Cc03_g05800	Long-chain-alcohol oxidase FAO1		-0.07	-2.32	-0.12	-2.75		-0.88	-0.18	-2.45
Cc06_g15960	Omega-6 fatty acid desaturase, endoplasmic reticulum		-0.06	-2.91		-2.68	-0.3	-2.54	-0.21	-1.45
Cc08_g12140	Peroxisomal acyl-coenzyme A oxidase 1			0.54		0.41		-0.13		-0.22
Cc11_g16630	Putative 2,4-dichlorophenol 6-monooxygenase			0.3	0.05	0.53		0.34	0.17	1.23
Cc01_g06760	Putative 3-hydroxybenzoate 6-hydroxylase 1		-0.17						0.15	

Genotype		Icatu				CL153			
CO2		380		700		380		700	
Temperature		IT	HI	IT	HI	IT	HI	IT	HI
Cc10_g16200	Putative 3-hydroxybenzoate 6-hydroxylase 1	0.07	1.35		1.36				
Cc10_g01240	Putative 3-hydroxybenzoate 6-hydroxylase 1						-1.33		
Cc11_g01620	Putative 6-hydroxynicotinate 3-monooxygenase					0.26	1.08	0.17	
Cc07_g10150	Putative FAD-linked oxidases family protein				0.49			0.19	1.52
Cc07_g14730	Putative FAD/NAD(P)-binding oxidoreductase family protein	-0.03		0.13	0.99	-0.27	-2.16	-0.18	-1.68
Cc10_g16040	Putative Reticuline oxidase		0.55			0.22	1.1	0.09	0.5
Cc07_g20340	Putative Reticuline oxidase-like protein	-0.04	-0.56	0.04	-0.83	-0.16	-1.18		-1.29
Cc09_g07170	Putative Reticuline oxidase-like protein	-0.22	-3.82		-3.15		-1.93		-0.52
Cc03_g15260	Putative Reticuline oxidase-like protein	-0.12	-1.26			-0.18	-1.17	-0.23	-1.22
Cc04_g11400	Putative Reticuline oxidase-like protein	0.14	-0.95	0.2		0.23	1.04	0.33	1.6
Cc09_g07190	Putative Reticuline oxidase-like protein	0.13		0.1				-0.41	-3.28
Cc07_g14180	Putative Reticuline oxidase-like protein	0.15		0.16		0.19	1.18	0.3	1.59
Cc00_g29360	Putative Reticuline oxidase-like protein	0.12			-2.39				
Cc07_g20360	Putative Reticuline oxidase-like protein	0.37	3	0.44	3.02	0.34	2.71	0.5	2.77
Cc09_g07390	Putative Reticuline oxidase-like protein							-1.13	-3.53
Cc03_g15270	Putative Reticuline oxidase-like protein							-1.52	-3.34
Cc06_g14630	Putative Uncharacterized protein Cbei_0202	0.03	0.47	0.09	0.84		0.26	0.11	0.95
Cc08_g11580	Putative Zeaxanthin epoxidase, chloroplastic	0.21	1.19	0.06	-0.42	0.41	2.31	0.39	1.71
Cc09_g07450	Reticuline oxidase-like protein	-0.05			0.41			-0.41	-2.07
Cc09_g07430	Reticuline oxidase-like protein	-0.06	-2.14		-0.93	-0.18	-0.99	-0.45	-2.97
Cc09_g07410	Reticuline oxidase-like protein	0.13	1.1	0.3	3.68	-0.12	-0.61	-0.48	-2.6
Cc00_g22750	Reticuline oxidase-like protein		-1.77		-2.74				
Cc00_g25380	Reticuline oxidase-like protein				-1.52	0.22	1.03		-0.54
Cc09_g07420	Reticuline oxidase-like protein				-1.34	0.26	1.32		

Genotype			Icatu				CL153			
CO_2			380		700		380		700	
Temperature			IT	HI	IT	HI	IT	HI	IT	HI
Cc05_g14750	DUF21 domain-containing protein At1g55930, chloroplastic	flavin adenine dinucleotide binding			0.05				0.06	0.36
Cc02_g29450	Glucose inhibited division protein A, putative		0.07		0.14	1.6			0.29	2.1
Cc07_g10140	Probable D-2-hydroxyglutarate dehydrogenase, mitochondrial				0.05	0.44			0.11	0.89
Cc09_g07200	Putative FAD-binding Berberine family protein			-4.08					-0.58	-4.45
Cc02_g29440	tRNA uridine 5-carboxymethylaminomethyl modification enzyme MnmG		0.12	0.97	0.17	1.44	0.07	0.69	0.2	1.51
Cc06_g01200	Acyl-coenzyme A oxidase 4, peroxisomal	oxidoreductase activity	-0.04				0.05			-0.47
Cc03_g01030	Dihydrolipoyl dehydrogenase		-0.05	-0.56	-0.06	-0.35		-0.64	-0.05	-1.11
Cc02_g34890	Dihydrolipoyl dehydrogenase		-0.18	-2.37	-0.27	-2.46	-0.09	-0.86	-0.16	-1.01
Cc03_g00430	Dihydrolipoyl dehydrogenase 1, mitochondrial			-0.73		-1.23	0.14	0.5	0.23	0.81
Cc02_g19680	FAD-linked sulfhydryl oxidase ERV1		-0.07	1.18		1.33	-0.13		-0.19	
Cc00_g00330	Flavin-containing monooxygenase FMO GS-OX-like 3			-0.81		-1.17			0.36	
Cc08_g12870	Flavin-containing monooxygenase FMO GS-OX-like 3						-0.07			
Cc02_g24320	Flavin-containing monooxygenase FMO GS-OX5							-0.97		-1.28
Cc02_g24290	Flavin-containing monooxygenase FMO GS-OX5							-1.59		
Cc01_g20250	Flavin-containing monooxygenase YUCCA10		0.17	2.27		1.49	0.31	1.6	0.09	0.68
Cc08_g08920	Flavin-containing monooxygenase YUCCA6		0.21							
Cc07_g06290	FMN-linked oxidoreductases superfamily protein			-0.52		-0.29	-0.06	-0.34		
Cc02_g01700	Glutamate synthase [NADH], amyloplastic		0.12	1.13		0.58	0.15	0.44	0.02	-0.18
Cc03_g07600	Glutathione reductase, chloroplastic (Fragment)		-0.03		-0.03				-0.05	-0.46
Cc01_g18820	Glutathione reductase, chloroplastic (Fragment)		-0.08	-1.12	-0.1	-1.67	-0.09	-1.12	-0.07	-1.32
Cc01_g04060	Linoleate 13S-lipoxygenase 2-1, chloroplastic		-0.1	-2.66	0.28	-1.02	-0.49	-2.64	-0.1	-0.72
Cc02_g13400	Linoleate 13S-lipoxygenase 3-1, chloroplastic		-0.73	-5.43	-0.73	-6.62				

**Genotype**		**Icatu**				**CL153**			
**CO2**		**380**		**700**		**380**		**700**	
**Temperature**		**IT**	**HI**	**IT**	**HI**	**IT**	**HI**	**IT**	**HI**
Cc00_g27370	Linoleate 13S-lipoxygenase 3-1, chloroplastic	-0.19		0.13	0.71	-0.65	-2.96	-0.4	
Cc00_g30760	Linoleate 13S-lipoxygenase 3-1, chloroplastic	-0.11	-0.83	0.09	0.4	-0.6	-3.03	-0.46	-0.85
Cc05_g01710	Linoleate 13S-lipoxygenase 3-1, chloroplastic					-0.6	-4.16		
Cc03_g03580	Linoleate 9S-lipoxygenase 5, chloroplastic	0.07	-0.58	0.11		0.08			0.64
Cc11_g16680	Lipoxygenase 6, choloroplastic	-0.04	-0.81	-0.03	-1.16	-0.09	-0.92	-0.1	-1.13
Cc11_g04070	Long-chain-alcohol oxidase FAO1		-1.32		-1.74			0.13	
Cc01_g18050	Long-chain-alcohol oxidase FAO2		-1.07	0.1	-0.9	-0.27	-2.57	-0.15	-1.97
Cc08_g06040	Long-chain-alcohol oxidase FAO4A	-0.25	-6.24	-0.34	-7.9				
Cc06_g05490	Monodehydroascorbate reductase		0.38	-0.06	-0.27		-0.39	0.05	
Cc08_g15200	Monodehydroascorbate reductase, chloroplastic	0.05		0.11	0.36	0.12	0.62	0.19	1.29
Cc06_g13220	NADPH--cytochrome P450 reductase	0.06		-0.14	-2.45	0.22	0.6	-0.11	-1.5
Cc10_g15930	NADPH--cytochrome P450 reductase	0.13	1.27	0.14	1.35	0.14	1.01	0.14	1.18
Cc07_g08880	NADPH-dependent diflavin oxidoreductase ATR3	-0.05				-0.12			
Cc01_g14330	Omega-3 fatty acid desaturase, endoplasmic reticulum		-2.2	-0.25	-4.37		-0.64		-0.64
Cc01_g05180	Omega-6 fatty acid desaturase, endoplasmic reticulum isozyme 2	0.11	-1.03	-0.02	-2.36	0.29	0.31	0.04	-1.76
Cc01_g05170	Omega-6 fatty acid desaturase, endoplasmic reticulum isozyme 2							-0.71	-3.47
Cc06_g15110	Probable monodehydroascorbate reductase, cytoplasmic isoform 2	0.17	1.14			0.27	1.43	0.11	0.67
Cc07_g02030	Probable monodehydroascorbate reductase, cytoplasmic isoform 2	0.19	1.29	0.06		0.26	1.3	0.12	0.5
Cc05_g00340	Probable polyamine oxidase 2	0.12	1.36	0.05	1.63	0.05	0.29	-0.07	
Cc06_g11400	Probable polyamine oxidase 5		-2.73		-3.72	0.26			
Cc04_g01940	Protein HOTHEAD	-0.39	-5.6	-0.27	-5.49	0.14		-0.1	-3.18
Cc08_g13880	Protein HOTHEAD	-0.07	-2.61	-0.3	-3.72	0.26	1	0.08	-1.32

Genotype		Icatu				CL153			
CO_2		380		700		380		700	
Temperature		IT	HI	IT	HI	IT	HI	IT	HI
Cc02_g03540	Protein HOTHEAD		-3.86		-4.35				-2.13
Cc09_g01850	Putative Abnormal spindle-like microcephaly-associated protein homolog	-0.16	-2.41	-0.16	-2.61				-2.94
Cc03_g01500	Putative Acyl-CoA dehydrogenase family member 10	0.04	1.17	0.1	1.41		0.28	0.07	0.7
Cc01_g20210	Putative Flavin-containing monooxygenase YUCCA10	0.31	3.56	0.09	2.05	0.34	1.99	0.15	1.26
Cc00_g00340	Putative Flavin-containing monooxygenase-like		2.98						
Cc08_g09200	Putative L-gulonolactone oxidase	0.22	-1.41	0.22	-1.77	0.35	1.43	0.17	1.35
Cc06_g07530	Putative Probable flavin-containing monooxygenase 1	-0.41	-8.2	-0.54	-7.82	-0.37	-4.78		-3.4
Cc00_g29350	Putative Reticuline oxidase	0.12	-1.32		-2.35			-0.5	-4.31
Cc00_g22730	Putative Reticuline oxidase				-2.13				
Cc09_g07180	Putative Reticuline oxidase-like protein		-2.07		-3.46			-0.55	-2.86
Cc09_g07460	Putative Reticuline oxidase-like protein		-2.83	0.14	-2.61	-0.06	-2.26	-0.08	-0.72
Cc09_g07380	Putative Reticuline oxidase-like protein							-1.45	-3.39
Cc09_g03720	Putative Riboflavin biosynthesis protein RibD			0.05				0.06	0.48
Cc07_g18910	Putative tRNA-dihydrouridine synthase A	-0.04		-0.06	-0.98	-0.05	-0.33	0.08	
Cc03_g07370	Putative Ubiquinone biosynthesis monooxygenase COQ6		0.33	0.08	0.62			0.1	0.58
Cc07_g08820	Putative uncharacterized protein	0.33	4.55		4.47		3.88		3.74
Cc09_g07440	Reticuline oxidase-like protein		0.69	-0.07				-0.47	-2.72
Cc03_g15290	Reticuline oxidase-like protein							-1.13	-4.88
Cc02_g20360	Squalene monooxygenase	-0.13	-3.32	-0.29	-4.39	-0.11	-1.82	-0.25	-3.49
Cc07_g07420	Squalene monooxygenase	-0.08	-2.12	0.13	-1.98	-0.16	-1.85	0.09	-1.56
Cc10_g07750	Squalene monooxygenase		-1.7	-0.17	-3.33	0.14		-0.23	-2.02
Cc07_g09450	Succinate dehydrogenase [ubiquinone] flavoprotein subunit 1, mitochondrial		0.82	-0.05	0.35		0.26		0.26
Cc07_g07740	tRNA-dihydrouridine(20) synthase [NAD(P)]-like	0.1	1.38	0.07	1.42		0.66		1.23

Genotype			Icatu				CL153			
CO_2			380		700		380		700	
Temperature			IT	HI	IT	HI	IT	HI	IT	HI
Cc02_g16820	tRNA-dihydrouridine(47) synthase [NAD(P)( )]-like		-0.05	-0.29			-0.13	-1.08	-0.09	-0.94
Cc07_g06010	Zeaxanthin epoxidase, chloroplastic		0.13			-1.51	0.25	0.85	0.18	0.2
Cc10_g07160	Cryptochrome-1	photoreceptor activity	0.11	0.93	0.13	1.05	0.1	0.79	0.16	1.19
Cc10_g00510	Acetolactate synthase 2, chloroplastic	transferase activity	0.03	0.36		0.46			0.05	0.58
Cc05_g13590	fatty acid desaturase A	N/A	0.3	2.25	-0.19	-1.9	0.58	3.16	0.19	0.45
Cc02_g06400	Omega-3 fatty acid desaturase, chloroplastic		-0.08	-3.66	-0.2	-5.39	0.12	-0.73	-0.07	-2.06
Cc06_g15100	Omega-6 fatty acid desaturase, chloroplastic			-0.32	-0.02	-0.55	0.13	0.75	0.15	1
**Cellular Respiration**										
Cc02_g17310	cytochrome BC1 synthesis	ATP binding	-0.36	-1.5	-0.18	-0.77				
Cc00_g04360	Putative cytochrome BC1 synthesis		-0.32	-2.66	-0.1	-1.54	-0.13	-1.59		-1.39
Cc05_g12050	cytochrome c oxidase 17	copper chaperone activity	0.09	1.38	0.07	1.25	0.12	0.75	0.08	0.61
Cc05_g05920	Cytochrome b6-f complex iron-sulfur subunit, chloroplastic	electron transfer activity	0.08		0.08	-0.27	0.18	0.83	0.27	1.07
Cc02_g23400	Cytochrome c		0.03	0.75		1.26	0.08	0.47		0.18
Cc04_g15830	Cytochrome c1, heme protein, mitochondrial		0.05	0.45	-0.04	-0.31			-0.04	-0.58
Cc09_g06180	Cytochrome c6, chloroplastic		0.07	0.46	0.07					
Cc07_g20970	Succinate dehydrogenase [ubiquinone] iron-sulfur subunit 2, mitochondrial		-0.05	-0.49		-0.62		-0.38	-0.06	-0.58
Cc06_g07030	D-lactate dehydrogenase [cytochrome], mitochondrial	FAD binding	0.06	0.6	0.1	0.59	0.04		0.2	0.44
Cc06_g13220	NADPH--cytochrome P450 reductase	flavin adenine dinucleotide binding	0.06		-0.14	-2.45	0.22	0.6	-0.11	-1.5
Cc10_g15930	NADPH--cytochrome P450 reductase		0.13	1.27	0.14	1.35	0.14	1.01	0.14	1.18
Cc07_g09450	Succinate dehydrogenase [ubiquinone] flavoprotein subunit 1, mitochondrial			0.82	-0.05	0.35		0.26		0.26

Genotype			Icatu				CL153			
**CO2**			**380**		**700**		**380**		**700**	
**Temperature**			**IT**	**HI**	**IT**	**HI**	**IT**	**HI**	**IT**	**HI**
Cc00_g20370	Putative Cytochrome c-type biogenesis protein CcmE	heme binding	0.07	0.86		0.86				0.7
Cc07_g01320	Cytochrome b-c1 complex subunit Rieske, mitochondrial	ion binding	0.11	0.66			0.11	1.05		0.51
Cc01_g17010	Cytochrome b5			-1.24	-0.06	-1.87	0.04	-0.51	-0.11	-1.41
Cc02_g39770	Cytochrome b5			-1.48	-0.1	-2.1	0.13			-0.65
Cc01_g15760	Cytochrome b561/ferric reductase transmembrane with DOMON related domain			-2.7	0.15	-2.54		-1.18	-0.15	-2
Cc07_g12990	Cytochrome c oxidase assembly protein ctaG		0.05	0.66	0.07	1.23	0.14	0.97	0.12	1.58
Cc06_g10460	Putative Cytochrome b5		0.07							
Cc04_g07670	Putative Cytochrome c-type biogenesis protein CcmH		0.19	2.85	0.13	2.64	0.26	2.48	0.25	2.3
Cc01_g15470	Cytochrome b-c1 complex subunit 8	oxidoreductase activity							0.06	
Cc11_g14980	Cytochrome b-c1 complex subunit Rieske-2, mitochondrial				-0.04				-0.05	-0.55
Cc02_g01060	Cytochrome b5 isoform 1		0.05							
Cc02_g09720	Cytochrome c oxidase assembly protein COX15			-0.91	-0.1	-1.26				-0.52
Cc02_g29800	Cytochrome c oxidase assembly protein COX15			0.78	0.19	1.87				
Cc04_g03950	Cytochrome c oxidase subunit 5b-2, mitochondrial			0.56	0.03	0.39		0.34	0.04	0.23
Cc07_g07610	Cytochrome c oxidase subunit 6a, mitochondrial			-0.51	-0.05	-0.91			-0.05	-0.89
Cc02_g36440	Cytochrome P450 704C1		0.08	0.95	0.08	0.85		0.63	0.07	0.95
Cc09_g09430	Cytochrome P450 710A1		0.31	2.45	0.22	1.69	0.43	3.67	0.39	2.43
Cc10_g09650	Cytochrome P450 716B2		-0.2	-0.82	-0.23	-1.67	-0.32	-1.22	-0.32	-1.84
Cc01_g00180	Cytochrome P450 716B2		0.25	2.42	0.09	-0.23	0.47	2.68	0.4	1.46
Cc06_g23800	Cytochrome P450 716B2			-3.82	-0.31	-3.68	-0.36	-4.44	-0.49	-4.95
Cc06_g23770	Cytochrome P450 716B2			-1.12	-0.1	-1.42	-0.05	-1.26	-0.15	-2.21
Cc11_g07610	Cytochrome P450 716B2				-0.58	-6.17				

Genotype		Icatu				CL153			
CO_2		380		700		380		700	
Temperature		IT	HI	IT	HI	IT	HI	IT	HI
Cc04_g11190	Cytochrome P450 71A1	-0.24	-2.28	-0.12	-2.52	-0.54	-4.17	-0.5	-3.67
Cc04_g11200	Cytochrome P450 71A1	-0.23	-3.2	-0.2	-3.9	0.12	-0.76	0.04	-1.58
Cc03_g10990	Cytochrome P450 71A2	-0.19	-1.32		-2.26			-1.7	-3.34
Cc04_g11250	Cytochrome P450 71A2	0.19	0.81	0.05	-0.93	0.28	1.07		
Cc00_g18530	Cytochrome P450 71A2	0.24	1.35		-1.21				
Cc03_g10910	Cytochrome P450 71A2	0.03		0.08	-0.22	0.16			
Cc00_g18520	Cytochrome P450 71A2	0.12		0.08	-0.97				
Cc00_g09210	Cytochrome P450 71A2			0.16		0.29	1.32		
Cc03_g15180	Cytochrome P450 71A2					0.92	5.6	0.56	2.41
Cc00_g18540	Cytochrome P450 71A4	0.16	1.35	0.12	0.22	0.39	2.18	0.46	2.17
Cc04_g11270	Cytochrome P450 71A8	0.03		0.15	0.61	-0.24	-0.89	0.13	0.91
Cc00_g28960	Cytochrome P450 71A8	0.1	0.97	0.31	2.5	-0.72	-3.91		
Cc00_g09200	Cytochrome P450 71A8					-0.31	-1.73		
Cc00_g33270	Cytochrome P450 71A8					-0.71	-5.81		
Cc05_g14770	Cytochrome P450 71D10	-0.19				-0.32	-2.27		
Cc05_g05080	Cytochrome P450 71D10	-0.27	-2.4			-0.2	-2.79	-0.35	-4.28
Cc08_g12110	Cytochrome P450 71D10	-0.38	-5.3			-0.73	-4.92	-0.46	-2.54
Cc01_g02610	Cytochrome P450 71D10	-0.27	-6.52	-0.13	-6.66	-0.67	-4.97	-0.53	-3.08
Cc05_g05110	Cytochrome P450 71D10	0.11	0.37		-0.9				
Cc05_g05090	Cytochrome P450 71D10							0.13	
Cc04_g03930	Cytochrome P450 734A1	-0.21	-4.93	-0.19	-3.42	-0.33	-2.18	-0.2	-2.43
Cc06_g08950	Cytochrome P450 734A1	-0.12	-3.18		-2.33	-0.4	-4.4	-0.28	-5.05
Cc04_g01230	Cytochrome P450 76C2	-0.14	-1.71	-0.14	-2.01	-0.06	-1.66	-0.27	-3.07
Cc02_g36410	Cytochrome P450 76C2	0.08		0.06	-0.49	0.24			-2.86
Cc02_g36400	Cytochrome P450 76C4	0.04	-0.34	0.03	-1.02		-0.84	0.1	-0.66

Genotype		Icatu				CL153			
CO2		380		700		380		700	
Temperature		IT	HI	IT	HI	IT	HI	IT	HI
Cc02_g10080	Cytochrome P450 77A3	-0.03	0.44	-0.16	0.98		-1.35	-0.14	-3.27
Cc11_g04730	Cytochrome P450 78A3	0.23	1.06	0.44	2.33	-0.11	-1.39	0.23	
Cc02_g39780	Cytochrome P450 78A3	0.26	-0.65	0.03	-3.26	0.38	1.41	0.09	-0.33
Cc11_g16860	Cytochrome P450 78A4	0.21					1.24	-0.42	
Cc07_g18490	Cytochrome P450 81D1	0.08	-0.24	-0.05	-1.72	0.19			-1.38
Cc00_g12590	Cytochrome P450 81D1		-1.04		-1.46	0.18		-0.14	-2.15
Cc10_g03650	Cytochrome P450 82A3				-0.83			0.21	
Cc10_g03630	Cytochrome P450 82A3						-1.59	-0.22	-1.89
Cc05_g01740	Cytochrome P450 82C4	0.16	0.68	0.07	-1.48			-0.14	-1.33
Cc05_g01750	Cytochrome P450 82G1				-2.43	0.22	1.23	0.1	0.66
Cc06_g22070	Cytochrome P450 83B1	-0.05	-3.25	-0.25	-6.48	0.54	2.06	0.14	-1.55
Cc07_g10360	Cytochrome P450 84A1	0.16			-0.64	0.05	-0.74	-0.07	-0.18
Cc00_g14180	Cytochrome P450 85A1	-0.22	-2.17		0.63	-0.16	-3.1	-0.16	-3.43
Cc02_g13800	Cytochrome P450 86A1								-2.16
Cc08_g14880	Cytochrome P450 86A2	-0.1	-3.89	-0.35	-6.3	0.16		-0.06	-2.84
Cc02_g38150	Cytochrome P450 86A2		-2.81	-0.29	-5.09	0.28	1.44		-2.84
Cc11_g02430	Cytochrome P450 86B1		-2.39		-1.95				-1.66
Cc00_g11910	Cytochrome P450 87A3	-0.35	-5.26	-0.21	-4.9				
Cc02_g06630	Cytochrome P450 90A1	0.11		0.23	0.6	0.2	1.26	0.24	0.56
Cc07_g09440	Cytochrome P450 90B1	-0.16	-1.18	-0.07	-0.36	-0.33	-1.79	-0.34	-1.67
Cc06_g16320	Cytochrome P450 93A1	0.17	2.69	0.43	4.22				
Cc06_g16330	Cytochrome P450 93A1		3.44						
Cc01_g18620	Cytochrome P450 94A1		2.89		1.56				
Cc01_g18610	Cytochrome P450 94A1					0.21		-0.26	
Cc05_g00020	Cytochrome P450 97B2, chloroplastic	-0.05		-0.03			-0.68	0.1	0.22

Genotype		Icatu				CL153			
CO2		380		700		380		700	
Temperature		IT	HI	IT	HI	IT	HI	IT	HI
Cc00_g08150	Cytochrome P450 98A2	0.23	-0.7	0.28	-1.13	0.16			0.61
Cc06_g20390	Cytochrome P450 98A3	0.14	1.35	0.07	0.29	0.21	1.23	0.05	0.27
Cc06_g21550	NADH-cytochrome b5 reductase 1	-0.07	-0.53	-0.08	-0.65		-0.28	-0.14	-0.67
Cc06_g08770	NADH-cytochrome b5 reductase-like protein		-0.27		-0.32		0.6	0.09	0.52
Cc00_g11030	Putative Cytochrome P450		2.51						
Cc02_g36450	Putative Cytochrome P450 704C1	0.1	1.18	0.11	0.81	-0.06	-0.84	0.06	
Cc03_g12610	Putative Cytochrome P450 704C1					-0.55	-3.61	-0.49	-3.27
Cc00_g24260	Putative Cytochrome P450 716B1	-0.39	-3.08	-0.22					
Cc10_g09620	Putative Cytochrome P450 716B2	-0.35	-3.05	-0.21	-3.1				-2.36
Cc04_g08820	Putative Cytochrome P450 716B2	0.12	0.81	0.06		0.24	1.46	0.14	0.42
Cc11_g16380	Putative Cytochrome P450 71A1	-0.14	-3.31		-2.72		-1.29		-2.1
Cc07_g16720	Putative Cytochrome P450 71A1		-0.44		-0.41	-0.12	-1.01	-0.06	-1.08
Cc09_g06410	Putative Cytochrome P450 71A1						-1.47		
Cc07_g15910	Putative Cytochrome P450 71A26	-0.29	-2.82		-1.97	-0.24	-3.06	-0.2	-3.03
Cc04_g11300	Putative Cytochrome P450 71A26	0.14	1.23	0.11		0.35	1.94	0.51	2.76
Cc07_g15870	Putative Cytochrome P450 71B10	-0.3			3.43	-0.49	-3.77	-0.25	-1.78
Cc07_g15900	Putative Cytochrome P450 71B10	-0.18	-1.6	0.27		-0.8	-4.57		
Cc07_g15920	Putative Cytochrome P450 71B10					-0.76	-5.12		
Cc07_g15890	Putative Cytochrome P450 71B10						-3.56		
Cc05_g05070	Putative Cytochrome P450 71D11 (Fragment)	0.06	0.86	0.11	0.91				0.78
Cc02_g25420	Putative Cytochrome P450 734A1	-0.44	-4.72	-0.21	-2.95	-0.24	-1.68		
Cc10_g03140	Putative Cytochrome P450 734A1	-0.42	-3.56				-1.24		
Cc02_g05010	Putative Cytochrome P450 734A1	-0.24	-2.65		-2.43		-1.01		
Cc09_g07590	Putative Cytochrome P450 734A1		-3.42		-4.38				
Cc10_g03120	Putative Cytochrome P450 734A1		0.67			-0.24	-1.48		

**Genotype**		**Icatu**				**CL153**			
**CO2**		**380**		**700**		**380**		**700**	
**Temperature**		**IT**	**HI**	**IT**	**HI**	**IT**	**HI**	**IT**	**HI**
Cc07_g19960	Putative Cytochrome P450 734A1		1.04		1.33				
Cc11_g11650	Putative Cytochrome P450 734A1					-0.15	-2.1		-1.78
Cc11_g11640	Putative Cytochrome P450 734A1						-1.68	0.16	
Cc01_g15550	Putative Cytochrome P450 734A6	-0.39	-3.9	-0.23	-4.56		-2.23	-0.34	-3.57
Cc02_g29220	Putative Cytochrome P450 750A1				-4.85			-0.51	-3.4
Cc06_g05070	Putative Cytochrome P450 76A2	0.17	0.87	0.25	0.4				
Cc06_g05060	Putative Cytochrome P450 76A2		1.78						
Cc08_g13150	Putative Cytochrome P450 76C1	-0.1	0.49	-0.09	0.4	-0.27	-2.59	-0.38	-2.49
Cc08_g03920	Putative Cytochrome P450 76C4		-1.98		-1.84	0.19			-1.72
Cc11_g01750	Putative Cytochrome P450 76C4			0.1	2.03				
Cc00_g19570	Putative Cytochrome P450 76C4				2.61				
Cc09_g01950	Putative Cytochrome P450 77A3	-0.31	-6.11	-0.53	-8.32	0.35	1.33		-2.52
Cc10_g05250	Putative Cytochrome P450 81D1	-0.06	0.37	0.26	2.6	-0.35	-1.87		
Cc01_g03760	Putative Cytochrome P450 81D1	0.18							
Cc10_g05360	Putative Cytochrome P450 81D1	0.11	1.71		0.88				-1.28
Cc10_g05380	Putative Cytochrome P450 81D1	0.14	3.01	0.16	3.02				-1.58
Cc01_g03750	Putative Cytochrome P450 81D1	0.2	0.96	0.11		0.32	1.23		-0.7
Cc00_g24670	Putative Cytochrome P450 81D1	0.08	0.43	0.2	0.43		1.15	0.57	4.12
Cc01_g16610	Putative Cytochrome P450 82A1 (Fragment)	-0.24		-0.09	-0.55	-0.39	-2.34	-0.49	-2.65
Cc00_g25660	Putative Cytochrome P450 82A1 (Fragment)	0.25							
Cc04_g10590	Putative Cytochrome P450 82A1 (Fragment)							-1.43	-3.52
Cc04_g10600	Putative Cytochrome P450 82A3		10.03		4.65	-0.4	-3.52	-1.34	-2.96
Cc10_g09170	Putative Cytochrome P450 82C4	0.26		0.06	-1.02	0.52	1.3	0.07	-2.28
Cc00_g30530	Putative Cytochrome P450 83B1	-0.26		-0.25				-0.88	-3.44
Cc06_g22430	Putative Cytochrome P450 83B1		-1.16						

Genotype			Icatu				CL153			
CO2			380		700		380		700	
Temperature			IT	HI	IT	HI	IT	HI	IT	HI
Cc06_g22050	Putative Cytochrome P450 83B1			0.65	0.3	2.49		-1.02	-0.57	-2.9
Cc06_g22450	Putative Cytochrome P450 83B1				-0.27				-0.46	-4.66
Cc07_g16350	Putative Cytochrome P450 86B1		-0.05	-1.81	-0.08	-1.94		-0.93		-2.92
Cc09_g02370	Putative Cytochrome P450 86B1		0.14	1.98	0.23	2.66				
Cc00_g05170	Putative Cytochrome P450 86B1			-0.75	0.12	-1.04	0.06		0.08	-0.5
Cc00_g35810	Putative Cytochrome P450 86B1				0.16					-0.74
Cc09_g05150	Putative Cytochrome P450 89A2		-0.12	-3.86		-3.35				
Cc02_g06570	Putative Cytochrome P450 89A9		0.02	0.43	-0.06	-0.61	-0.15	-0.56	-0.06	-0.4
Cc01_g09500	Putative Cytochrome P450 94A1		-0.44	-6.14					-1.21	-3.56
Cc06_g05540	Putative Cytochrome P450 94A1		-0.21	-3.11	-0.35	-4.04				
Cc06_g20240	Putative Cytochrome P450 94A1		0.03			-0.82	0.07	0.41	0.14	0.61
Cc01_g05670	Putative Cytochrome P450 94A1			-0.55	0.08	-0.49		0.21	0.26	1.04
Cc01_g15540	Putative cytochrome P450, family 714, subfamily A, polypeptide 1				-0.23	-5.05				
Cc07_g16990	Putative cytochrome P450, family 76, subfamily C, polypeptide 4			-2.71		-3.96			-1.2	-4.29
Cc00_g01610	Succinate dehydrogenase cytochrome b560 subunit				-0.27	1.76				1.87
Cc02_g18220	cytochrome b6f complex subunit (petM), putative	protein binding	-0.03	-1.32	-0.04	-1.77	0.03	-0.94	-0.04	-1.61
Cc04_g11340	Putative Uncharacterized sufE-like protein slr1419	transferase activity	0.08		-0.12	-1.55	0.06	-0.17	-0.11	-0.79
Cc06_g20970	Succinate dehydrogenase [ubiquinone] iron-sulfur subunit 2, mitochondrial		0.11	1.96		1.08		0.4	-0.07	
Cc11_g01730	Cytochrome b-c1 complex subunit 7	N/A	0.13	1.31	0.12	1.13	0.13	0.62	0.17	0.65
Cc01_g09260	Cytochrome b-c1 complex subunit 9		-0.04						-0.06	-0.74
Cc09_g09250	Cytochrome b561/ferric reductase transmembrane protein family		0.09	1.38	0.04	1.35	0.12	1.31	0.06	1.54

Genotype			Icatu				CL153			
**CO2**			**380**		**700**		**380**		**700**	
**Temperature**			**IT**	**HI**	**IT**	**HI**	**IT**	**HI**	**IT**	**HI**
Cc02_g39350	Cytochrome b561/ferric reductase transmembrane with DOMON related domain		0.19	-2	0.17	-3.09	0.19		-0.05	
Cc02_g17920	Cytochrome c biogenesis protein CCS1, chloroplastic		-0.08	-0.43		0.39	-0.18	-0.81	0.08	0.93
Cc04_g02780	cytochrome c oxidase 19-2		0.08	1.15	0.07	0.87		0.91		0.79
Cc02_g34860	Cytochrome c oxidase biogenesis protein Cmc1-like		0.09	0.64	0.08	0.36			0.1	0.31
Cc07_g08920	Cytochrome c oxidase biogenesis protein Cmc1-like		0.07	0.93	0.09	0.87				
Cc07_g01900	Cytochrome c oxidase subunit 5C		0.26	1.8	0.2			2.27	0.29	2
Cc01_g21450	Cytochrome c oxidase subunit 5C-2		-0.03		-0.07	-0.31		-0.2	-0.03	-0.31
Cc06_g14120	Cytochrome c oxidase subunit 6b-1		-0.05	-0.95	-0.06	-1.35				-0.88
Cc10_g06750	Cytochrome c oxidase subunit 6b-1		0.03	0.88		0.99			-0.06	-0.33
Cc08_g01750	Cytochrome c oxidase subunit 6b-3								-0.17	-1.17
Cc05_g12670	Cytochrome c-type biogenesis ccda-like chloroplastic protein 2		-0.12	-1.98		-1.6	-0.12	-1		-0.5
Cc01_g09610	cytochrome-c oxidases;electron carriers		-0.03	-0.4	-0.04	-0.37	-0.05	-0.66	-0.11	-0.95
Cc05_g09720	Putative Cytochrome B561-related protein			-4.78						-2.04
Cc05_g09710	Putative Cytochrome b561/ferric reductase transmembrane protein family			-4.37	-0.21	-6.26	0.45	2.04		
Cc02_g04670	ubiquinol-cytochrome C chaperone family protein		-0.05	-0.51			-0.12	-0.44		0.61
**Malate dehydrogenase activity**										
Cc03_g01030	Dihydrolipoyl dehydrogenase	flavin adenine dinucleotide binding	-0.05	-0.56	-0.06	-0.35		-0.64	-0.05	-1.11
Cc03_g00430	Dihydrolipoyl dehydrogenase 1, mitochondrial			-0.73		-1.23	0.14	0.5	0.23	0.81
Cc02_g00480	Aluminum-activated malate transporter 4	NAD binding	0.03	0.89		1.01		0.42	-0.05	
Cc02_g19200	D-3-phosphoglycerate dehydrogenase, chloroplastic		-0.24	-2.96	-0.23	-2.02	-0.49	-3.91	-0.43	-3.34
Cc02_g32120	Histidinol dehydrogenase, chloroplastic								0.1	0.37

Genotype			Icatu				CL153			
CO2			380		700		380		700	
Temperature			IT	HI	IT	HI	IT	HI	IT	HI
Cc02_g10620	Malate dehydrogenase [NADP], chloroplastic		0.03		-0.06	-1.13	0.1	0.23	0.08	-0.17
Cc02_g37470	NAD-dependent malic enzyme 59 kDa isoform, mitochondrial		-0.08	-0.78			-0.13	-1.02		-0.59
Cc02_g25700	NAD-dependent malic enzyme 62 kDa isoform, mitochondrial		-0.12	-0.69	-0.08	-0.53	-0.09	-0.8	-0.07	-0.78
Cc06_g10370	NADP-dependent malic enzyme		-0.14	-0.91	0.1		-0.36	-2.31	-0.03	-0.52
Cc08_g09860	NADP-dependent malic enzyme		-0.06	-0.55	-0.07	-0.77			-0.07	-0.89
Cc11_g00600	Dihydroorotate dehydrogenase (quinone), mitochondrial	oxidoreductase activity		0.79		1.5	-0.09		-0.1	0.49
Cc06_g15830	Hypothetical protein					-0.98				
Cc04_g16570	Malate dehydrogenase, chloroplastic		0.05			0.21	0.13	0.68	0.04	
Cc02_g24520	Malate dehydrogenase, cytoplasmic		-0.1	-2.78		-2.8				-2.12
Cc03_g05530	Malate dehydrogenase, cytoplasmic				-0.02	-0.44	0.11	0.23		-0.58
Cc02_g20400	Malate dehydrogenase, glyoxysomal		0.08		-0.03	-1.73	0.22	0.6	0.15	-0.27
Cc04_g02590	NADP-dependent malic enzyme, chloroplastic		0.05		-0.1	-0.68	0.1	0.47	-0.16	-1.13
Cc04_g00540	Phytoene dehydrogenase, chloroplastic/chromoplastic			-0.27	-0.03	-0.68		0.19	0.11	0.45
Cc01_g07600	Putative Bifunctional aspartokinase/homoserine dehydrogenase			-0.54		-1			0.2	
Cc10_g04290	Putative dihydroflavonol-4-reductase		0.18	3.03	0.2	3.81	0.1	2.04	0.11	2.43
Cc02_g04270	Putative Succinate dehydrogenase subunit 3			-0.52	-0.04	-0.6		-0.58	-0.09	-1.13
Cc00_g01610	Succinate dehydrogenase cytochrome b560 subunit				-0.27	1.76				1.87
Cc04_g01660	Dihydrolipoyllysine-residue acetyltransferase component 3 of pyruvate dehydrogenase complex, mitochondrial	transferase activity				0.2				
Cc04_g10080	Malate synthase, glyoxysomal			4.02	0.1	3.02			-0.23	
Cc09_g00800	Tonoplast dicarboxylate transporter	transporter activity		-0.34	-0.13	-2.05	-0.05	-1.27	-0.32	-2.7

**Genotype**			**Icatu**				**CL153**			
**CO2**			**380**		**700**		**380**		**700**	
**Temperature**			**IT**	**HI**	**IT**	**HI**	**IT**	**HI**	**IT**	**HI**
Cc08_g02450	Aluminum-activated malate transporter 12	N/A	-0.04	-2.05		-1.72		-0.94		-0.73
Cc01_g08500	Aluminum-activated malate transporter 2			-0.83	0.16	-1.24	0.14	0.55		-0.88
Cc04_g07240	Aluminum-activated malate transporter 9		-0.05	-0.38	-0.1	-0.89	0.07	-0.32	-0.03	-1.12
Cc08_g01340	Omega-amidase NIT2		-0.07	-0.7	-0.04	-0.78	-0.09	-0.46	0.06	
Cc08_g15510	Putative Aluminum-activated malate transporter 9		-0.14	-2.73		-4.85	-0.41	-3.44		-2.56
**Pyruvate kinase activity**										
Cc02_g04080	Adenylate kinase	ATP binding				-0.81		-0.51		-0.99
Cc06_g07370	Adenylate kinase, chloroplastic		0.08		0.05	-0.56	0.1	0.27	0.16	0.19
Cc06_g03450	Adenylate kinase, chloroplastic		0.07	1.67	0.07	2.36		0.64	0.05	1.06
Cc06_g23010	Adenylyl-sulfate kinase 1, chloroplastic									-0.52
Cc07_g07070	Adenylyl-sulfate kinase, chloroplastic				-0.09	-0.43		0.85	0.08	0.6
Cc07_g01220	Aspartokinase 1, chloroplastic		0.06	0.45	0.09	0.97	0.08	0.94	0.13	1.23
Cc10_g08630	Bifunctional aspartokinase/homoserine dehydrogenase, chloroplastic (Fragment)		-0.04	-0.5					0.08	0.6
Cc02_g39660	Bifunctional fucokinase/fucose pyrophosphorylase		0.03	0.37	0.07	0.81			0.06	0.9
Cc06_g00520	CBL-interacting serine/threonine-protein kinase 14		0.22	0.58		-1.56	0.57	3.06	0.09	0.3
Cc06_g15160	CBL-interacting serine/threonine-protein kinase 6			-0.54	-0.08	-1.94	0.11	0.37		-0.98
Cc07_g19430	Cyclin-dependent kinase E-1		0.05	0.62	0.08	0.45			0.15	0.7
Cc04_g15010	Diacylglycerol kinase 1						-0.05			
Cc05_g06060	diacylglycerol kinase 5		0.03	-0.93	0.07	-1.28	-0.05	-0.86	-0.07	-1.37
Cc00_g15600	Mevalonate kinase		0.04			-0.4	0.32	1.4	0.2	0.38
Cc05_g09440	Mitogen-activated protein kinase 10		-0.04	-0.89	-0.09	-1.22			-0.14	-0.99
Cc03_g02990	Mitogen-activated protein kinase 16		0.13	0.82	0.08	0.71	0.17	1.23		0.35
Cc05_g10580	Mitogen-activated protein kinase 19		0.09	0.48	-0.03		0.25	1.01	0.04	-0.17
Cc01_g11790	Mitogen-activated protein kinase 3		-0.15	-1.27	-0.07	-1.53	-0.05	-1	-0.17	-1.87

**Genotype**		**Icatu**				**CL153**			
**CO2**		**380**		**700**		**380**		**700**	
**Temperature**		**IT**	**HI**	**IT**	**HI**	**IT**	**HI**	**IT**	**HI**
Cc08_g15160	Mitogen-activated protein kinase 4	-0.12	1.58	-0.09	2.1	-0.22	-0.34	-0.14	1.02
Cc07_g10000	Mitogen-activated protein kinase 7			0.06		-0.07	-0.56		-0.43
Cc04_g06490	Mitogen-activated protein kinase 9	-0.11	-0.94	-0.13	-1.65		-0.46	-0.2	-1.45
Cc03_g14980	Mitogen-activated protein kinase homolog MMK1		0.32		0.3	0.05	0.47		0.24
Cc00_g17870	Mitogen-activated protein kinase homolog MMK2	0.09	1.61		1.3	0.19	1.73		1.17
Cc01_g11870	Mitogen-activated protein kinase homolog NTF6	-0.1	-1.49		-2.03			-0.18	-1.28
Cc03_g04850	P-loop containing nucleoside triphosphate hydrolases superfamily protein			0.13	0.98	-0.5	-3.25	-0.44	-2.08
Cc01_g18690	P-loop containing nucleoside triphosphate hydrolases superfamily protein						-1.86		-2.43
Cc04_g14200	Pantothenate kinase 2	-0.07	-0.91	-0.04	-1.05	-0.11	-0.98		-0.69
Cc11_g15230	Pantothenate kinase 2					-0.1	-0.3		
Cc02_g02100	Plastidial pyruvate kinase 2	-0.16	-1.28	-0.18	-1.45	-0.11	-1.49	-0.19	-2.13
Cc02_g02110	Plastidial pyruvate kinase 2	-0.17	-1.32	-0.2	-1.28	-0.11	-1.75	-0.14	-1.98
Cc09_g02340	Probable adenylate kinase 1, chloroplastic	0.11	1.17					-0.16	-0.97
Cc04_g16270	Probable adenylate kinase isoenzyme 6							-0.06	
Cc01_g00290	Probable pyruvate kinase, cytosolic isozyme	-0.05	-0.37	-0.08	-0.64	-0.14	-1.26	-0.12	-1.25
Cc06_g02180	Protein kinase superfamily protein	-0.03		-0.05	-0.43	-0.07			
Cc11_g00240	Putative Homoserine kinase	0.06	0.93		0.56		-0.4	-0.08	-0.41
Cc03_g07080	Putative Hydroxyethylthiazole kinase		0.64		0.62			0.12	0.55
Cc00_g04720	Putative Mitogen-activated protein kinase kinase 6	-0.12	-0.98	-0.05	-1.08	-0.09	-0.79		-0.61
Cc06_g13770	Putative Probable diacylglycerol kinase 3	-0.04	-1.02	-0.05	-1.17	-0.12	-0.96	-0.1	-1.27
Cc02_g10330	Putative Pyruvate kinase, cytosolic isozyme	-0.09	-0.89	-0.09	-1.15	-0.12	-1.23	-0.09	-1.29
Cc06_g05020	Putative Pyruvate kinase, cytosolic isozyme	-0.08		-0.08		-0.06	-1.18	-0.08	-1.36
Cc04_g10720	Putative Thiamin pyrophosphokinase 1		0.56	-0.07	0.64	-0.14		-0.07	0.46

Genotype			Icatu				CL153			
CO2			380		700		380		700	
Temperature			IT	HI	IT	HI	IT	HI	IT	HI
Cc03_g00110	Putative uncharacterized protein						0.08	1	0.14	1.27
Cc11_g12440	Putative Uncharacterized protein MJ0044			-0.51		-0.49	-0.1	-0.58		
Cc01_g18850	Putative uridine kinase C227.14			-0.8		-0.72	-0.13	-0.55		-0.43
Cc04_g07920	Putative Uridine-cytidine kinase C		-0.04		-0.03		-0.1	-0.33	-0.06	-0.35
Cc11_g11180	Putative Uridine-cytidine kinase C		-0.11	-0.63						
Cc11_g08540	Putative Uridine-cytidine kinase C		-0.07							
Cc06_g05030	Putative Uridylate kinase		0.03	0.65		0.77				
Cc02_g20130	Pyruvate kinase isozyme A, chloroplastic		-0.29	-3.99	-0.44	-5.44	-0.17	-1.37	-0.31	-4.52
Cc03_g04490	Pyruvate kinase isozyme A, chloroplastic						0.09	0.22	0.07	
Cc08_g02330	Pyruvate kinase isozyme G, chloroplastic			-0.26	-0.03	-1.09	0.04	-0.18	0.05	-0.43
Cc11_g00360	Pyruvate kinase, cytosolic isozyme		-0.1	-1.03	-0.11	-0.92	-0.14	-0.84	-0.09	-0.71
Cc04_g09680	Pyruvate kinase, cytosolic isozyme								0.33	1.04
Cc03_g02730	Pyruvate, phosphate dikinase, chloroplastic		0.32	2.65	0.08		0.43	2.48	0.44	1.93
Cc08_g01880	UMP/CMP kinase		-0.07	-1.52	0.08	-0.79	-0.13	-1.33		-0.6
Cc02_g25770	UMP/CMP kinase		-0.07	-1.48	-0.04	-1.09		-0.95		-0.91
Cc07_g17890	Uridylate kinase				0.15	0.92			0.06	0.84
Cc02_g30660	Light-inducible protein CPRF2	DNA binding	0.05	1.01	0.04	1.12			0.05	0.63
Cc04_g05920	Dual specificity protein phosphatase PHS1	hydrolase activity	-0.1	-1.17	-0.12	-1.14	-0.12	-0.95	-0.09	-0.7
Cc02_g01450	PP2A regulatory subunit TAP46		0.04	1.84		2.1			-0.09	
Cc07_g08070	Probable fructokinase-4		-0.04	-1.31	-0.11	-2.07		-0.54	0.09	-0.33
Cc04_g09560	Pyruvate dehydrogenase E1 component subunit beta, mitochondrial		-0.03	-0.28		-0.22				-0.25
Cc02_g38470	C-terminal binding protein AN	NAD binding	0.08	0.45	-0.05	0.37	0.12	0.75		
Cc04_g09990	Adenylate kinase B	oxidoreductase activity		-0.43		-0.46	0.09			-0.69
Cc02_g39940	Pyruvate dehydrogenase E1 component subunit alpha			-1.04		-1.25		-1.62	-0.1	-2.08

Genotype			Icatu				CL153			
CO_2			380		700		380		700	
Temperature			IT	HI	IT	HI	IT	HI	IT	HI
Cc11_g14000	Pyruvate dehydrogenase E1 component subunit alpha, mitochondrial		0.04			0.31	0.09	0.29	0.06	
Cc05_g10200	Pyruvate dehydrogenase E1 component subunit beta		-0.11	-1.83	-0.28	-1.93	0.11	0.67		
Cc03_g15970	Pyruvate dehydrogenase E1 component subunit beta		0.07	0.46	0.08	0.59	0.09	0.82	0.18	1.18
Cc11_g17430	Pyruvate dehydrogenase E1 component subunit beta, mitochondrial			0.56	-0.2	-2.18		-0.86	0.24	
Cc06_g04020	Adenosine kinase 2	protein binding	-0.11	-3.2	-0.18	-4.21		-1.66	-0.2	-2.06
Cc01_g04370	Adenosine kinase 2						0.31	2.54	0.51	3.18
Cc01_g19450	Cyclin-P3-1		-0.08	-2.66	-0.2	-3.85	0.2		-0.11	-1.91
Cc05_g05150	Cyclin-U1-1		0.12	0.91	0.21	1.32		0.38	0.16	1.13
Cc02_g24650	Cyclin-U2-2		-0.1	-1.37	-0.26	-4.19			-0.24	-3.62
Cc02_g25260	Cyclin-U4-1		-0.43	-7.08	-0.51	-8.84	-0.42	-4.62	-0.66	-3.18
Cc01_g10560	phosphatidate cytidylyltransferase family protein		-0.03		-0.06	0.56				0.43
Cc02_g17780	Phosphatidylinositol 4-kinase alpha		-0.06	-0.52	-0.06	-0.26	-0.06	-0.31	-0.04	-0.18
Cc07_g18300	Phosphatidylinositol 4-kinase beta 1			0.49		0.86		0.67		0.85
Cc02_g33470	Predicted protein			-2.27		-1.91		-1.16		-1.17
Cc02_g25540	Protein of unknown function (DUF581)		-0.2	-3.23	-0.19	-3.84	0.07	-1.37	-0.09	-1.38
Cc02_g37230	Protein-ribulosamine 3-kinase, chloroplastic								0.18	0.86
Cc09_g03080	Putative Translational activator GCN1		-0.05		-0.03		-0.09	-0.27	0.05	0.59
Cc04_g01660	Dihydrolipoyllysine-residue acetyltransferase component 3 of pyruvate dehydrogenase complex, mitochondrial	transferase activity				0.2				
Cc02_g22780	Putative Thiamine biosynthesis bifunctional protein ThiED					0.44		0.41	0.12	1.01
Cc02_g00150	Putative Uncharacterized sugar kinase slr0537		0.12	0.8	0.12	0.58	0.24	1.52	0.17	1.23
Cc09_g00970	Brain protein 44	N/A							-0.05	

Genotype		Icatu				CL153			
CO2		380		700		380		700	
Temperature		IT	HI	IT	HI	IT	HI	IT	HI
Cc04_g09980	Brain protein 44-like protein	-0.09		-0.16	-0.87	-0.11	-1.28	-0.29	-2.24
Cc02_g01610	Putative Polyubiquitin	0.3	3.72	0.36	4.11	0.58	4.71	0.92	5.74
Cc02_g39410	Putative Polyubiquitin-B	-0.04		-0.06	0.28		-0.19	-0.11	-0.43
Cc08_g16190	Putative Polyubiquitin-B	0.12							
Cc03_g13260	UPF0041 protein R07E5.13			-0.05	-0.54		-0.34	-0.08	-0.36
